# SFK Inhibition Suppresses EBV-Encoded BART miRNAs and Induces Apoptosis in EBV-Positive Gastric Epithelial Cells

**DOI:** 10.3390/cancers18071082

**Published:** 2026-03-26

**Authors:** Yuxin Liu, Zolzaya Tumurgan, Aung Phyo Wai, Moushumi Akter, Afifah Fatimah Azzahra Ahmad Wadi, Yoichi Mizukami, Masami Wada, Shunpei Okada, Daisuke Niino, Takayuki Murata, Hisashi Iizasa, Hironori Yoshiyama

**Affiliations:** 1 Department of Microbiology, Faculty of Medicine, Shimane University, 89-1 Enya, Izumo 693-8504, Shimane, Japan; yul604@pitt.edu (Y.L.); zolzaya.t@purec.jp (Z.T.); phyothethein@gmail.com (A.P.W.); m249611@med.shimane-u.ac.jp (M.A.); m229401@med.shimane-u.ac.jp (A.F.A.A.W.); mawada@med.shimane-u.ac.jp (M.W.); s.okada@med.shimane-u.ac.jp (S.O.); 2 Department of Microbiology and Molecular Genetics, UPMC Hillman Cancer Center, University of Pittsburgh, Pittsburgh, PA 15219, USA; 3 PuREC Co., Ltd., 89-1 Enya-cho, Izumo City 693-0021, Shimane, Japan; 4 Center for Gene Research, Yamaguchi University, Ube 755-8505, Yamaguchi, Japan; mizukami@yamaguchi-u.ac.jp; 5 Department of Pathology, Faculty of Medicine, Shimane University, 89-1 Enya, Izumo 693-8504, Shimane, Japan; dniino@med.shimane-u.ac.jp; 6 Department of Virology, Fujita Health University School of Medicine, 1-98 Dengakugakubo, Kutsukake-cho, Toyoake 470-1192, Aichi, Japan; tmurata@fujita-hu.ac.jp

**Keywords:** Epstein–Barr virus, apoptosis, gastric cancer, *Bam*H I A rightward transcripts, microRNA, Dasatinib, Src family kinase

## Abstract

**Background/Objectives:** Epstein–Barr virus (EBV) is associated with a subset of gastric carcinomas characterized by latency programs that promote survival of infected cells. EBV-encoded *Bam*H I A rightward transcript (BART) microRNAs contribute to apoptosis resistance in infected epithelial cells. This study investigated whether dasatinib, a Src family kinase (SFK) inhibitor, selectively targets EBV-positive gastric epithelial cells and examined the molecular mechanisms underlying this effect. **Methods:** EBV-positive and EBV-negative gastric epithelial cell models were analyzed to evaluate cell viability, apoptosis induction, signaling pathways, and viral gene regulation. BART miRNA expression was quantified by RT-qPCR, and promoter activity was examined using luciferase reporter assays. Downstream target gene expression was analyzed at both the transcript and protein levels. Recombinant EBV lacking BZLF1 or LMP2A was used to assess the contributions of lytic activation and LMP2A-associated signaling. **Results:** Dasatinib preferentially reduced viability and induced apoptosis in EBV-positive gastric epithelial cells compared with EBV-negative counterparts. Treatment suppressed phosphorylation of Src and ERK and reduced expression of the anti-apoptotic proteins BCL-xL and MCL1. Apoptosis was also observed in cells infected with LMP2A-deficient EBV, suggesting that the effect cannot be fully explained by inhibition of LMP2A-associated signaling. Dasatinib inhibited BART miRNA promoter activity and reduced pri-, pre-, and mature miR-BART levels, accompanied by increased expression of pro-apoptotic target genes including *CASZ1a*, *OCT1*, *ARID2*, *TP53INP1*, and *DAB2*. In parallel, dasatinib suppressed BZLF1 promoter activity without evidence of lytic reactivation. **Conclusions:** Dasatinib promotes apoptosis in EBV-positive gastric epithelial cells in association with coordinated suppression of SFK signaling and EBV-encoded BART miRNA expression, accompanied by derepression of pro-apoptotic cellular genes. These findings reveal a previously underappreciated vulnerability of EBV-positive epithelial cells and suggest that targeting host kinase signaling pathways that regulate viral microRNAs may represent a potential therapeutic strategy for EBV-associated malignancies.

## Simple Summary

Epstein–Barr virus (EBV) infects most people worldwide and is associated with several malignancies, including a subset of gastric cancers. In EBV-infected epithelial cells, the virus produces BART microRNAs (miRNAs) that suppress apoptosis and support the survival of infected cells. We previously showed that inhibition of BART miRNA promoter activity is associated with increased apoptosis in EBV-infected cells. In the present study, we demonstrate that dasatinib, a clinically used tyrosine kinase inhibitor, suppresses BART miRNA promoter activity and reduces BART miRNA expression. This reduction is accompanied by increased expression of several pro-apoptotic cellular genes and preferential apoptosis in EBV-infected gastric epithelial cells. Importantly, these effects occurred without evidence of productive viral replication. These findings identify a previously unrecognized link between dasatinib and viral microRNA regulation and suggest that targeting EBV-encoded miRNAs may represent a potential therapeutic strategy for EBV-associated cancers.

## 1. Introduction

Epstein–Barr virus (EBV) infects over 90% of people. Primary infection is asymptomatic in childhood but often causes mononucleosis in adolescence. After primary infection, most people continue an asymptomatic latent infection, but in some, persistently infected B or epithelial cells become tumorigenic [1].

EBV-associated gastric carcinoma (EBVaGC) accounts for 5–10% of all gastric cancers and arises from a single clone of persistently EBV-infected cells [2]. Unlike EBV-negative gastric cancers, EBVaGC cells show high DNA methylation, express immune checkpoint molecules programmed cell death1-ligand 1 (PD-L1) and PD-L2 and often lack p53 mutations [3]. The EBVaGC cells display type I latent infection, with sustained expression of EBV-associated nuclear antigen1 (EBNA1), latent membrane protein 2A (LMP2A), EBV-encoded small RNA (EBER)s, and *Bam*H I A rightward transcript (BART)s. Notably, BART introns encode ~40 non-coding microRNAs (miRNAs) that suppress apoptosis and immune responses, promoting tumorigenesis [4]. In EBV-infected gastric epithelial cells, BART miRNAs suppress the expression of various pro-apoptotic genes, such as castor zinc finger 1a (CASZ1a), octamer-binding transcription factor 1 (OCT1), tumor protein p53-inducible nuclear protein 1 (TP53INP1), AT-rich interaction domain 2 (ARID2), and disabled homolog 2 (DAB2) [5,6,7]. We reported that an inhibitor of BART miRNA promoter activity induced apoptosis in EBVaGC cells [5].

Given its distinct genetic profile, histone deacetylase inhibitors such as vorinostat and romidepsin have been reported to induce lytic infection and selective cell death in EBV-positive gastric epithelial cells [5,8,9,10]. However, during the malignant transformation of EBV-associated tumors, deletions often occur in the viral genome [11]. In such cases, induction of lytic infection may fail to trigger viral replication, limiting tumor cell lysis [12]. Therefore, therapeutic strategies other than lytic induction are needed to selectively target EBV-infected tumor cells.

Dasatinib is a tyrosine kinase inhibitor targeting breakpoint cluster region-Abelson murine leukemia (BCR-ABL), used in the treatment of chronic myeloid leukemia [13]. In addition to BCR-ABL, dasatinib inhibits phosphorylation of Src family kinases (SFKs), ephrin type-A receptor 2 (EphA2), and c-kit [14,15]. Because of the broader kinase-inhibitory activity, dasatinib is also being investigated for use in BCR-ABL-negative cancers such as non-Hodgkin lymphoma and metastatic breast cancer [16,17]. Dasatinib suppressed lymphoma development in LMP2A transgenic mice and inhibited the proliferation of EBV-transformed lymphoblastoid cell lines (LCLs) in vitro [18]. However, it showed no inhibitory effect on LCL growth in immunodeficient mice [19]. On the other hand, EBV-positive gastric epithelial cells do not express anti-apoptotic factors such as LMP1, EBNA2, or EBNA3 [2], but strongly express BART miRNA. Thus, we hypothesized that dasatinib might suppress the proliferation of EBV-positive gastric epithelial cells through inhibiting EBV miRNAs’ expression [5]. We therefore examined its effects using EBV-infected and EBV-uninfected gastric epithelial cells. As a result, dasatinib induced stronger apoptosis in EBV-infected gastric epithelial cells than in uninfected gastric epithelial cells.

## 2. Materials and Methods

### 2.1. Cells and Viruses

Several human gastric carcinoma cells were used. AGS cells harboring wild-type *TP53* were obtained from the American Type Culture Collection (ATCC, Manassas, VA, USA). MKN28 cells carrying mutant *TP53* were obtained from the JCRB Cell Bank at the National Institutes of Biomedical Innovation, Health and Nutrition, Japan [20]. The *TP53* mutation in MKN28 cells was verified by Sanger sequencing [5]. YCCEL1 cells were kindly provided by Dr. Suk Kyong Lee at The Catholic University of Korea [21].

Each cell line was infected with a recombinant Akata EBV strain in which the enhanced green fluorescent protein (eGFP) and neomycin-resistance genes were inserted into the viral thymidine kinase locus without altering viral infectivity or replication capacity (eGFP-EBV) [22]. To establish *Bam*H I Z leftward fragment 1 (BZLF1) deficient (KO) EBV–positive AGS cells, BZLF1 KO Akata cells were transiently transfected with a BZLF1-expression plasmid, and the produced virions were used to infect AGS cells [23]. AGS cells infected with LMP2A KO EBV were prepared as previously described [24].

All cells were maintained in RPMI-1640 medium (Sigma-Aldrich, St. Louis, MO, USA) supplemented with 10% fetal bovine serum (FBS; Thermo Fisher Scientific, Waltham, MA, USA), 100 U/mL penicillin, and 100 μg/mL streptomycin (Nacalai, Kyoto, Japan) in a 37 °C, 5% CO_2_ incubator. EBV infection was maintained by the addition of 500 μg/mL G-418 (Promega, Madison, WI, USA).

### 2.2. Cell Proliferation Assay

Cells (6.25 × 10^3^ cells/well) were seeded into 96-well plates and treated the following day with dasatinib (Selleck Chemicals, Houston, TX, USA), ponatinib (Selleck Chemicals), saracatinib (Selleck Chemicals), or src inhibitor-1 (Medchem Express, Monmouth Junction, NJ, USA) dissolved in dimethyl sulfoxide (DMSO; Nacalai). The final DMSO concentration was adjusted to 0.1%. After 48 h, 10 μL of CCK-8 solution (DOIJNDO, Kumamoto, Japan) was added to 100 μL of medium. Absorbance at 450 nm was measured using a DTX880 spectrophotometer (Beckman Coulter, Brea, CA, USA). Cell viability was calculated relative to DMSO controls. Half-maximal inhibitory concentrations (IC_50_) were determined using GraphPad Prism software (v10.0.3; GraphPad Software, Boston, MA, USA).

### 2.3. Apoptosis Assay

Early apoptosis was assessed using the RealTime-Glo Annexin V Apoptosis Assay (Promega). Cells (6.25 × 10^3^ cells/well) were seeded in 96-well plates and treated the following day with dasatinib dissolved in DMSO. After 48 h, 100 μL of 2 × Detection Reagent (containing Annexin V NanoBiT substrate, CaCl_2_, Annexin V-SmBiT, and Annexin V-LgBiT) was added to 100 μL of medium. Apoptosis was measured using a GloMax Navigator luminometer (Promega).

### 2.4. Quantitative Polymerase Chain Reaction (qPCR)

Cells (1.1 × 10^6^ cells/10-cm dish) were treated with 0.1% DMSO or 2.5 μM dasatinib for 48 h. Genomic DNA was extracted using the GeneElute Mammalian Genomic DNA Miniprep Kit (Sigma-Aldrich). Primers targeting the EBV *BamH I W* region (F: 5′-CCCAA CACTCCACCACACC-3′, R: 5′-TCTTAGGAGGCTGTCCGAGG-3′) and human *glyceraldehyde 3-phosphate dehydrogenase (GAPDH)* (F: 5′-TGTGCTCCCACTCCTGTTT C-3′, R: 5′-CCTAGTCCCAGGGCTTTGATT-3′), as well as dual-quenched probes [*Bam*H I W: 5′-FAM/CACACACTA/ZEN/CACACACCCACCCGTCTC/IBFQ-3′ and GAPDH: 5′-FAM/CGGTCACAA/ZEN/TCTCACGC/IBFQ-3′] were purchased from Integrated DNA Technologies (IDT, Coralville, IA, USA). qPCR was performed using SsoAdvanced Universal Probe Supermix (Bio-Rad Laboratories, Hercules, CA, USA) on a CFX Connect Real-Time PCR Detection System (Bio-Rad) [25]. *GAPDH* served as an internal control.

### 2.5. Reverse Transcription–qPCR (RT-qPCR)

Cells (1.1 × 10^6^ cells/10-cm dish) were treated with 0.1% DMSO or 2.5 μM dasatinib for 48 h. Total RNA was extracted using ISOGEN (Nippon Gene, Tokyo, Japan), and complementary DNA (cDNA) synthesis was carried out with SuperScript III reverse transcriptase (Thermo Fisher Scientific). Gene expression was quantified using primers obtained from IDT targeting BART (F: 5′-GGCTGTTCCTGAACGACGAG-3′, R: 5′-CTATAGGCGCATCCTGCTGA-3′), BZLF1 (F: 5′-TCCGACTGGGTCGTGGTT-3′, R: 5′-GCTGCATAAGC TTGATAAGCATTC-3′), *Bam*H I R leftward fragment 1 (BRLF1) (F: 5′-GGCCCAAAAAT TGCAGATGT-3′, R: 5′-CCCACGGGCGAGAATG-3′), *Bam*H I A leftward fragment 5 (BALF5) (F: 5′-TAGGGCC GTCAAAGTTG-3′, R: 5′-ACCTGCGAAGACATAGAG-3′), *Bam*H I L leftward fragment 1 (BLLF1) (F: 5′-GTCAGTACACCATCCAGAGCC-3′, R: 5′-TTGGTAGAGAGCCTTCGTATG-3′), B-cell lymphoma-extra-large (BCL-XL) (F: 5′-AACTCTTCCGGGATGGGGTA-3′, R: 5′-CGACTGAAGAGTGAGCCCAG-3′), induced myeloid leukemia cell differentiation protein-1 (MCL1) (F: 5′-CTTCGGGAGCAGGCCAC-3′, R: 5′-GTTACGCCGTCGCTGAAAAC-3′), C-terminal Src Kinase (CSK) (F: 5′-GGTCAGC GACTTTGGTCTCA-3′, R: 5′-TCCGAAACTCCACACGTCAG-3′), GAPDH (F: 5′-AATC CCATCACCATCTTCCA-3′, R: 5′-TGGACTCCACGACGTACTCA-3′). RT-qPCR was performed using SsoAdvanced Universal SYBR Green Supermix (Bio-Rad) on the CFX Connect system under the following conditions: 98 °C for 2 min, followed by 40 cycles of 95 °C for 10 s and 60 °C for 30 s. *GAPDH* expression served as the internal reference.

### 2.6. Luciferase Assay

For transfection, 1 × 10^5^ cells were transfected with 100 ng of a vector expressing firefly luciferase under the control of the BART promoter [5] or the *BZLF1* promoter [26] and 1 ng of pGL4.75 (Promega), which expresses Renilla luciferase, using Lipofectamine™ 2000 (Thermo Fisher). Three hours after transfection, 2.5 μM dasatinib was added to the cells. Luciferase activity was measured 48 h post-transfection using the Dual-Glo^®^ Luciferase Assay (Promega) and a GloMax Navigator luminometer (Promega), and firefly luciferase values were normalized to Renilla luciferase activity.

### 2.7. Western Blotting

Cells (1.1 × 10^6^ cells/10-cm dish) were treated with 0.1% DMSO or 2.5 μM dasatinib for 48 h and lysed in RIPA buffer (Thermo Fisher Scientific) supplemented with protease inhibitors (cOmplete mini; Sigma-Aldrich) and phosphatase inhibitors (PhosSTOP; Sigma-Aldrich). Protein samples (5 μg) were resolved on 7.5–15% SDS–PAGE gels and transferred to PVDF membranes (Merck Millipore, Brea, CA, USA). Membranes were blocked with Block ACE (KAC, Amagasaki, Japan) or PhosphoBlocker Reagent (Cell Biolabs, San Diego, CA, USA). Primary antibodies included phospho-c-Src (Tyr416) (D49G4, Cell Signaling Technology (CST), Danvers, MA, USA), c-Src (36D10, CST), extracellular signal-regulated kinases 1/2 (ERK1/2) (w15133B, Biolegend, San Diego, CA, USA), phospho-ERK1/2 (Thr202/Tyr204) (4B11B69, Biolegend), cleaved-caspase-3 (Asp175) (5A1E, CST), Lyn (C13F9, CST), phospho-Lyn (Tyr507) (#2731, CST), Syk (D3Z1E, CST), phospho-Syk (Tyr525/526) (C87C1, CST), and GAPDH (EPR16891, Abcam, Cambridge, UK) Horseradish peroxidase-conjugated anti-rabbit, anti-rat, or anti-mouse IgG secondary antibodies were used. Signals were visualized with Immobilon reagents (Merck Millipore) and detected on X-ray film. GAPDH served as a loading control.

### 2.8. Doxycycline-Inducible LMP2A Overexpression System

The pTRIPZ vector (Horizon Discovery, Waterbeach, UK) was digested with *Xba* I and *Cla* I, and the TetR–TurboRFP fragment was cloned into pBluescript KS II. The pcDNA3-3 × FLAG-LMP2A plasmid was provided by Dr. Teru Kanda (Tohoku Medical and Pharmaceutical University). The *LMP2A* coding region fused to a 3 × FLAG tag was amplified using specific primers (F: 5′-GCACCGGTCGCCACCATGGACTACAAAGACCATGACGGTG-3′, 5′-GTCGACGGTTATCGATTATACAGTGTTGCGATATGGGGTC　G-3′), and KOD One polymerase (TOYOBO, Osaka, Japan). The TurboRFP region in pBluescript was replaced with 3 × FLAG-LMP2A using specific primers (F: 5′-GGTCCGAGGTTCTAGACGAGTTTACTCCCTATCA-3′, 5′-TCAAACAAACTATCG AT TATACAGTGTTGCGATATGG-3′) and In-Fusion Snap Assembly (Takara Bio, Shiga, Japan). The TetR–3 × FLAG-LMP2A cassette was then inserted into *Xba I*-*Cla I*–digested pTRIPZ.

Lentiviral particles were generated in HEK293T cells using TransIT-Lenti (Takara Bio) and a packaging mix (Horizon Discovery). AGS cells were infected with pTRIPZ or pTRIPZ–3 × FLAG-LMP2A lentivirus at a multiplicity of infection (MOI) 1 and treated with 2.5 μM doxycycline (TaKaRa Bio) for 2 days (5 × 10^5^ cells/well in 6-well plates). LMP2A expression was confirmed by anti-FLAG immunoblotting.

### 2.9. RNA Sequencing (RNA-Seq)

Total RNA from drug-treated or untreated EBV-infected or uninfected cells was extracted using ISOGEN. mRNA was purified with the NEB Next Poly(A) mRNA Magnetic Isolation Module (New England Biolabs, Ipswich, MA, USA). CDNA libraries were prepared using the NEB Next Ultra II RNA Library Prep Kit and NEB Nextplex Oligos (New England Biolabs). Indexed libraries were pooled and sequenced (75 bp paired-end reads) using an Illumina NextSeq platform (Illumina, San Diego, CA, USA). Reads were analyzed using CLC Genomics Workbench (v8.01; Qiagen, Hilden, Germany), and pathway analysis was performed using Ingenuity Pathway Analysis (IPA; Qiagen).

### 2.10. Statistical Analysis

Differences between two independent groups were evaluated using the Mann–Whitney U test. Data are presented as mean ± standard deviation (SD). Statistical significance was defined as *p* < 0.05.

## 3. Results

### 3.1. Dasatinib Preferentially Suppresses Proliferation and Induces Apoptosis in EBV-Positive Gastric Epithelial Cells

**Figure 1:**
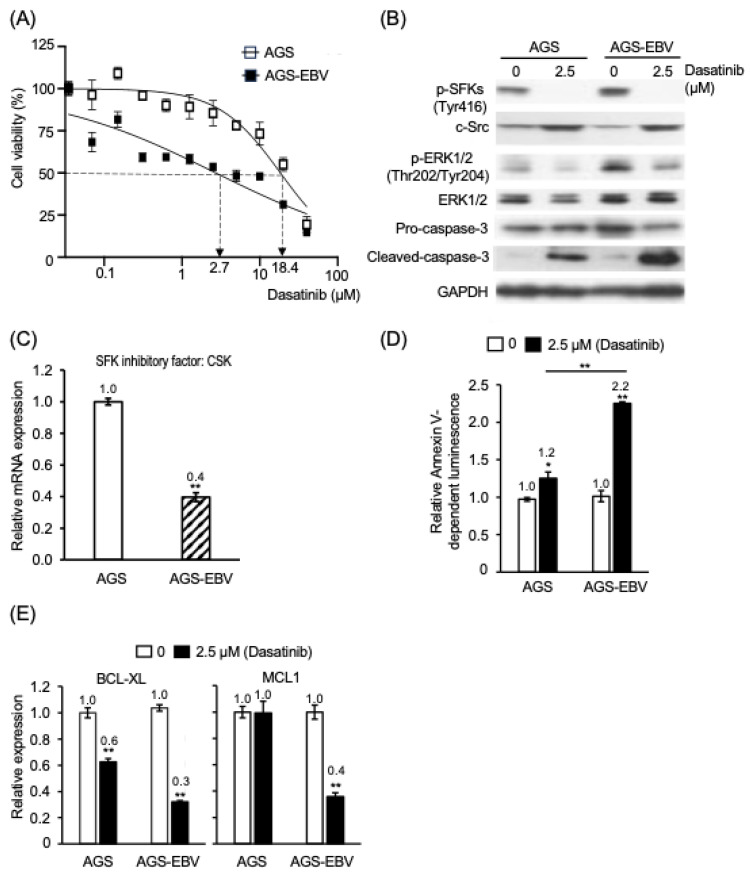
Dasatinib preferentially reduces viability and promotes early apoptosis in EBV-positive gastric epithelial cells. (**A**) Gastric epithelial AGS cells with or without persistent EBV infection were treated with increasing concentrations of dasatinib, and cell viability was assessed in a dose-dependent manner. Open squares indicate AGS cells, and closed squares indicate AGS-EBV cells. Viability of DMSO-treated controls was defined as 100%. (**B**) Expression of c-Src, phosphorylated Src family kinases (p-SFKs; Tyr416), phosphorylated ERK1/2 (p-ERK1/2; Thr202/Tyr204), total ERK1/2, pro-caspase-3, and cleaved caspase-3 following treatment with 2.5 μM dasatinib. Protein levels were analyzed by Western blotting with GAPDH as loading control. Representative blots from three independent experiments are shown. The uncropped western blot figures are presented in Supplementary Materials. (**C**) Relative mRNA expression of *CSK*, a negative regulator of SFKs, in AGS and AGS-EBV cells. White bars represent AGS cells, and hatched bars represent AGS-EBV cells. Expression levels were quantified by RT–qPCR, normalized to GAPDH, and expressed relative to AGS cells (set to 1). (**D**) Early apoptosis following 48 h of treatment with 2.5 μM dasatinib was assessed using Annexin V luminescence assay. Signals are expressed relative to untreated controls (set to 1). White bars indicate vehicle controls; black bars indicate dasatinib-treated cells. (**E**) mRNA expression of anti-apoptotic genes *BCL-XL* and *MCL1* following treatment with 2.5 μM dasatinib. White bars represent DMSO-treated controls, and black bars represent dasatinib-treated cells. Expression levels were quantified by RT–qPCR, normalized to GAPDH, and expressed relative to DMSO-treated controls (set to 1). Analyses were performed 48 h after treatment. Data are presented as mean ± SD from three independent experiments. *: *p* < 0.05, **: *p* < 0.01.

The IC_50_ values of dasatinib for p53-wild-type AGS cells and AGS-EBV cells were 18.4 μM and 2.7 μM, respectively (Figure 1A). Similarly, the IC_50_ values for TP53-mutant MKN28 cells and MKN28-EBV cells were 18.7 μM and 4.6 μM, respectively (Supplementary Figure S1A). In addition, the IC_50_ value for EBV-positive YCCEL1 cells, established from a patient with EBV-associated gastric cancer, was 4.0 μM (Supplementary Figure S1B). Notably, the IC_50_ values observed in EBV-positive cell lines (AGS-EBV, MKN28-EBV, and YCCEL1) were comparable (2.7 μM, 4.6 μM, and 4.0 μM, respectively). Consistent with these findings, the IC_50_ values of saracatinib for AGS and AGS-EBV cells were 31.3 μM and 8.4 μM, respectively (Supplementary Figure S2A), whereas those of src inhibitor 1 were 39.7 μM and 20.3 μM, respectively (Supplementary Figure S2B). Collectively, these results indicate that dasatinib and other tyrosine kinase inhibitors suppress the proliferation of EBV-positive gastric epithelial cells more effectively than that of EBV-negative cells.

Previous studies have shown that dasatinib inhibits SFKs, which activate ERK1/2, in addition to BCR-ABL, EphA2, and c-kit [14,15,27,28]. AGS-EBV cells exhibited higher levels of phosphorylated SFKs (p-SFKs) and phosphorylated ERK1/2 (p-ERK) than AGS cells (Figure 1B). Treatment with dasatinib (2.5 µM for 48 h) reduced p-c-SFK and p-ERK levels in AGS-EBV cells to levels comparable to those observed in AGS cells (Figure 1B). Under the same conditions, expression of *CSK*, a negative regulator of SFKs, was approximately 40% lower in AGS-EBV cells than in AGS cells (Figure 1C). These findings indicate that dasatinib suppresses SFK activation and downstream ERK signaling in EBV-positive cells.

Dasatinib treatment increased early apoptosis by approximately 1.2-fold in AGS cells and 2.2-fold in AGS-EBV cells (Figure 1D). Consistently, cleaved caspase-3 levels were higher in AGS-EBV cells than in AGS cells following treatment (Figure 1B). Moreover, dasatinib reduced expression of the anti-apoptotic gene *BCL-XL* by ~40% in AGS cells and ~70% in AGS-EBV cells (Figure 1E). Whereas *MCL1* expression was unchanged in AGS cells, it decreased by ~60% in AGS-EBV cells (Figure 1E). In line with these observations, mRNA profiling analysis revealed activation of the apoptotic pathway in dasatinib-treated AGS-EBV cells (Table 1). Taken together, these results suggest that dasatinib suppresses SFK signaling and is associated with enhanced apoptotic responses in EBV-positive cells compared with EBV-negative cells in a p53-independent manner.

### 3.2. Dasatinib Enhances Apoptosis in EBV-Positive Gastric Epithelial Cells Independently of Viral Lytic Reactivation

**Figure 2:**
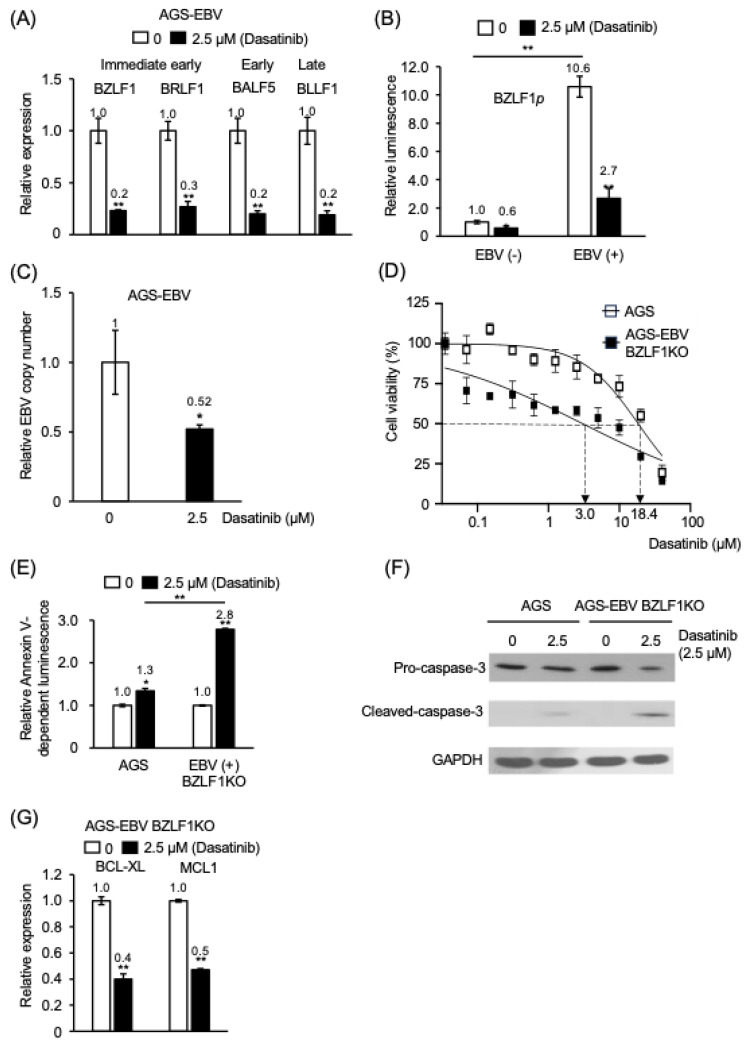
Dasatinib treatment is associated with preferential cell death in EBV-positive cells without evidence of productive lytic reactivation. (**A**) Expression of EBV immediate-early (*BZLF1*), early (*BRLF1* and *BALF5*), and late (*BLLF1*) genes in AGS-EBV cells following dasatinib treatment. (**B**) *BZLF1* promoter activity in AGS cells and AGS-EBV cells after dasatinib treatment. (**C**) Relative EBV genome copy number per cell in dasatinib-treated AGS-EBV cells, normalized to GAPDH as an internal control. (**D**) Cell viability of AGS cells and AGS-EBV BZLF1-knockout (BZLF1 KO) cells treated with increasing concentrations of dasatinib. (**E**) Early apoptosis following dasatinib treatment was assessed by Annexin V–based luminescence in AGS and AGS-EBV BZLF1KO cells. (**F**) Pro-caspase-3 and cleaved caspase-3 levels following dasatinib treatment, analyzed by Western blotting with GAPDH as a loading control. The uncropped western blot figures are presented in Supplementary Materials. (**G**) mRNA expression of anti-apoptotic genes *BCL-XL* and *MCL1* in dasatinib-treated AGS-EBV BZLF1 KO cells. Open bars indicate untreated controls; filled bars indicate treatment with 2.5 μM dasatinib. Expression values are presented relative to untreated controls (set to 1) after normalization to internal standards. Data are presented as mean ± SD from independent experiments. *: *p* < 0.05, **: *p* < 0.01.

Induction of the EBV lytic cycle in infected cells is known to trigger apoptosis [2]. To examine whether dasatinib-associated apoptosis in EBV-positive cells is linked to lytic reactivation, we first quantified EBV lytic gene expression in AGS-EBV cells. Compared with untreated cells, dasatinib-treated AGS-EBV cells exhibited marked suppression of lytic gene expression: the immediate-early genes *BZLF1* and *BRLF1*, the early gene *BALF5*, and the late gene *BLLF1* were reduced by approximately 80%, 70%, 80%, and 80%, respectively (Figure 2A). Similarly, in MKN28-EBV cells, dasatinib reduced expression of *BZLF1*, *BRLF1*, *BALF5*, and *BLLF1* by 71%, 67%, 49%, and 87%, respectively (Supplementary Figure S3A).

Because the *BZLF1* promoter is a key regulator of EBV lytic gene expression, we next examined its activity using a reporter assay. Basal *BZLF1* promoter activity was 10.6-fold higher in AGS-EBV cells than in AGS cells (Figure 2B). Treatment with dasatinib (2.5 µM for 48 h) reduced *BZLF1* promoter activity by 40% in AGS cells, whereas a substantially greater reduction (75%) was observed in AGS-EBV cells (Figure 2B). In parallel, the EBV genome copy number in AGS-EBV cells decreased to 52% of untreated levels following dasatinib treatment (Figure 2C). A comparable reduction was observed in MKN28-EBV cells, in which viral genome copy number decreased to 60% of untreated levels (Supplementary Figure S3B).

Because *BZLF1* encodes a viral transcription factor essential for initiation of the EBV lytic cycle [2], lytic reactivation does not occur in cells infected with EBV BZLF1 knockout (KO). Notably, the IC_50_ value of dasatinib in AGS cells infected with EBV BZLF1 KO was 3.0 μM, representing a 6.1-fold lower value than that observed in EBV-negative AGS cells (18.4 μM), indicating strong growth suppression despite the absence of lytic induction (Figure 2D). Furthermore, dasatinib increased early apoptosis by approximately 2.8-fold in EBV BZLF1 KO–positive cells compared with EBV-negative cells (Figure 2E) and was associated with increased cleaved caspase-3 levels (Figure 2F). In addition, dasatinib reduced expression of the anti-apoptotic genes *BCL-XL* and *MCL1* by 60% and 50%, respectively, in EBV BZLF1 KO–positive cells (Figure 2G).

Collectively, these findings indicate that dasatinib suppresses EBV lytic gene expression while promoting apoptotic responses in EBV-positive gastric epithelial cells, supporting the interpretation that its pro-apoptotic activity occurs independently of viral lytic reactivation.

### 3.3. Dasatinib Induces Apoptosis in EBV-Positive Gastric Epithelial Cells Through Mechanisms Independent of LMP2A Signaling

**Figure 3:**
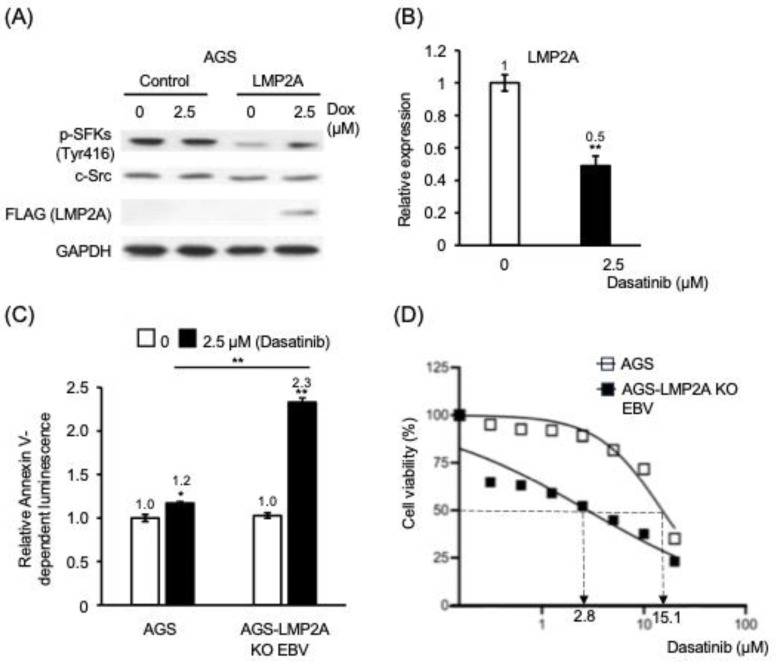
Preferential apoptosis of EBV-positive cells following dasatinib treatment is not fully attributable to inhibition of LMP2A-associated signaling. (**A**) Phosphorylation of SFKs (Tyr416) in AGS cells with or without inducible LMP2A expression. Cells were treated with doxycycline (Dox) and/or dasatinib as indicated. Total c-Src, FLAG-tagged LMP2A, and GAPDH are shown as controls. The uncropped western blot figures are presented in Supplementary Materials. (**B**) Relative LMP2A expression in EBV-positive cells following dasatinib treatment. (**C**) Early apoptosis following dasatinib treatment in AGS cells and AGS-EBV LMP2A-knockout (LMP2A KO) cells, assessed using Annexin V–based luminescence assay. (**D**) Sensitivity to dasatinib in AGS cells and AGS-EBV LMP2A KO cells determined by cell viability assays. Open bars or symbols indicate untreated controls; filled bars or symbols indicate treatment with 2.5 μM dasatinib. Values are presented relative to untreated controls (set to 1) after normalization to internal standards. Data represent mean ± SEM from independent experiments. *: *p* < 0.05, **: *p* < 0.01.

Previous studies in B lymphocytes have shown that dasatinib suppresses c-Src phosphorylation induced by LMP2A signaling [18]. To determine whether LMP2A exerts a similar effect in gastric epithelial cells, we induced LMP2A expression in AGS cells using a doxycycline-inducible system. Induction of LMP2A expression resulted in increased phosphorylation of SFKs, confirming activation of SFK signaling in this model (Figure 3A).

In persistently infected AGS-EBV cells, treatment with 2.5 µM dasatinib reduced LMP2A protein levels to approximately 50% of those in untreated cells (Figure 3B). This observation raised the possibility that reduced LMP2A expression might contribute to dasatinib-associated cell death in EBV-positive cells. To directly test this hypothesis, AGS cells persistently infected with EBV lacking LMP2A (LMP2A KO) were treated with 2.5 µM dasatinib. Notably, dasatinib treatment increased apoptosis by approximately 2.3-fold compared with untreated controls (Figure 3C).

Consistent with this finding, the IC_50_ value of dasatinib in AGS-EBV LMP2A KO cells was 2.8 μM, representing a 5.4-fold increase in drug sensitivity compared with EBV-negative AGS cells (IC_50_ = 15.1 μM) (Figure 3D). Together, these findings suggest that dasatinib-induced apoptosis in EBV-positive gastric epithelial cells cannot be fully explained by inhibition of LMP2A-mediated signaling.

### 3.4. Dasatinib Suppresses EBV BART miRNA Expression in EBV-Positive Gastric Epithelial Cells

EBV-encoded BART miRNAs have been reported to exert anti-apoptotic effects in infected cells [4]. In our previous study, we showed that dasatinib suppresses BART miRNA promoter activity [5]. Consistent with this observation, another SFK inhibitor, ponatinib, was also observed to suppress BART promoter activity (Supplementary Figure S4A) and reduce expression of BART transcripts in AGS-EBV cells (Supplementary Figure S4B), supporting a link between SFK signaling and BART transcriptional regulation. Based on these findings, we examined the relationship between BART miRNA expression and the expression of their reported target genes in EBV-positive cells.

**Figure 4:**
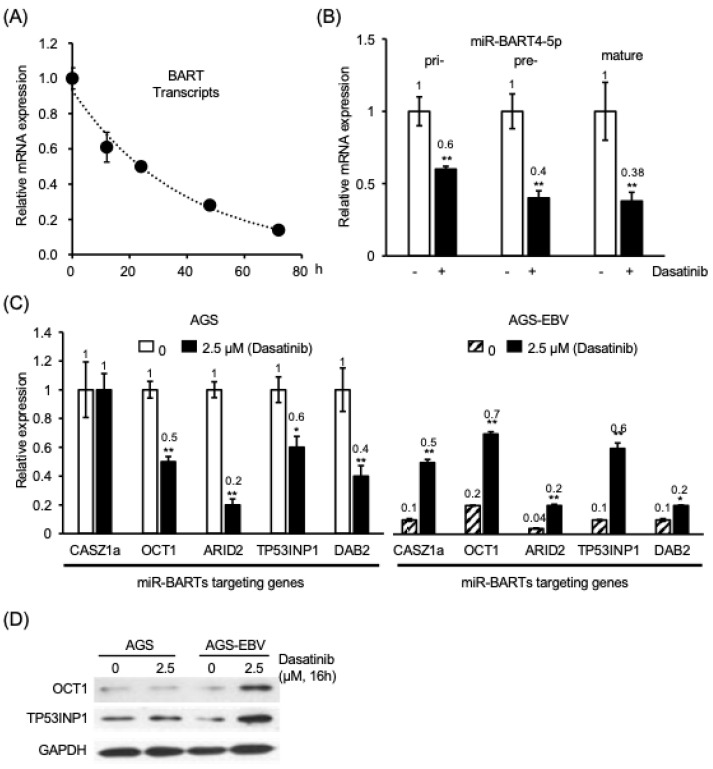
Dasatinib treatment is associated with reduced BART miRNA expression and modulation of reported miR-BART target genes in EBV-positive cells. (**A**) Time-course analysis of BART transcript levels in EBV-positive cells following dasatinib treatment. Relative mRNA levels were quantified by RT–qPCR and normalized to internal controls. (**B**) Expression of pri-miR-BART4, pre-miR-BART4, and mature miR-BART4-5p in EBV-positive cells following dasatinib treatment, determined by RT–qPCR. (**C**) Expression of representative cellular genes reported to be targeted by EBV miR-BARTs in AGS cells (left) and AGS-EBV cells (right) after dasatinib treatment. (**D**) Protein levels of OCT1 and TP53INP1, reported targets of miR-BARTs, in AGS and AGS-EBV cells treated with dasatinib for 16 h, analyzed by Western blotting with GAPDH as a loading control. Open bars indicate untreated controls; filled bars indicate treatment with 2.5 μM dasatinib. Expression values were normalized to internal standards and are presented relative to untreated controls (set to 1). The uncropped western blot figures are presented in Supplementary Materials. Data represent mean ± SEM from independent experiments. *: *p* < 0.05, **: *p* < 0.01.

Treatment with 2.5 µM dasatinib reduced BART transcript levels in AGS-EBV cells in a time-dependent manner (Figure 4A). Consistently, in AGS-EBV cells treated with 2.5 µM dasatinib, the expression levels of pri-, pre-, and mature miR-BART4-5p were reduced by 40%, 60%, and 62%, respectively, compared with untreated cells (Figure 4B). Basal expression of apoptosis-associated genes reported to be targets of miR-BARTs—*CASZ1a*, *OCT1*, *ARID2*, *TP53INP1*, and *DAB2*—was markedly lower in AGS-EBV cells than in EBV-negative AGS cells, with reductions of 70%, 80%, 90%, 70%, and 70%, respectively (Supplementary Figure S5A). Similarly, MKN28-EBV cells exhibited reduced expression of *CASZ1a*, *OCT1*, *ARID2*, and *DAB2* by 60%, 30%, 30%, and 60%, respectively, compared with EBV-negative MKN28 cells (Supplementary Figure S5B).

In EBV-negative AGS cells, dasatinib treatment was associated with reduced expression of the apoptosis-inducing genes *OCT1*, *ARID2*, *TP53INP1*, and *DAB2* by 50%, 80%, 40%, and 60%, respectively (Figure 4C, left). In contrast, in EBV-positive AGS-EBV cells, dasatinib treatment was associated with increased expression of *CASZ1a*, *OCT1*, *ARID2*, *TP53INP1*, and *DAB2* by 5.0-, 3.5-, 5.0-, 6.0-, and 2.0-fold, respectively (Figure 4C, right). A similar pattern was observed in dasatinib-treated MKN28-EBV cells, in which expression of *CASZ1a*, *OCT1*, *ARID2*, and *DAB2* increased by 2.1-, 1.9-, 1.4-, and 2.5-fold, respectively (Supplementary Figure S5C). Furthermore, increased protein expression of OCT1 and TP53INP1 was detected in AGS-EBV cells 16 h after dasatinib treatment, whereas no comparable increase was observed in AGS cells (Figure 4D).

Taken together, these findings indicate that dasatinib suppresses EBV-encoded BART miRNA expression, thereby relieving repression of pro-apoptotic cellular genes in EBV-positive cells.

## 4. Discussion

Activation of SFK signaling enhances the expression of the anti-apoptotic proteins BCL-XL and MCL1 via ERK1/2 activation, thereby suppressing apoptosis [29,30]. In EBV-positive gastric epithelial cells, corneal epithelial cells, and normal oral epithelial cells, the EBV latent genes *LMP1* and *LMP2A* have been reported to activate SFKs [31,32,33]. Persistent EBV infection is therefore proposed to contribute to sustained SFK signaling activity, potentially protecting host cells from apoptosis induced by virus-associated cellular stress.

Most compounds previously reported to selectively eliminate EBV-positive cells act by inducing the viral lytic cycle, and their antitumor efficacy has been demonstrated almost exclusively in cell lines harboring wild-type TP53 [8,9,10]. However, a subset of EBVaGCs carries mutant *TP53* and exhibits marked resistance to apoptosis [3]. Moreover, induction of EBV lytic infection leads to expression of the viral protein BNRF1, which destabilizes host chromosomes [34]. Consequently, suboptimal antitumor treatment may permit tumor cells that have acquired additional genetic alterations during lytic induction to survive and proliferate, thereby promoting malignant progression. In contrast, dasatinib treatment was associated with preferential apoptosis in EBV-positive cells, independent of the presence or absence of the viral lytic initiator gene *BZLF1* (Figure 2D). Furthermore, dasatinib also induced cell death in *TP53*-mutant MKN28 cells without detectable activation of canonical *TP53*-dependent markers, indicating that the observed apoptotic response does not appear to require intact TP53 signaling under the experimental conditions tested.

Dasatinib has previously been reported to induce apoptosis-independent cell death in LCLs [19]. However, because EBV-negative LCLs were not available in that study, it remained unclear whether dasatinib preferentially induces cytotoxicity in EBV-positive cells. In contrast, in the present study using EBV-positive epithelial cells, dasatinib treatment was accompanied by early apoptotic markers, including caspase-3 activation, preferentially in EBV-positive cells (Figure 1B). These findings indicate that biological responses to SFK inhibition vary depending on cellular context and EBV latency status.

This apparent discrepancy may be attributable to differences in EBV latency programs. LCLs exhibit type III latency and express multiple latent viral genes, including *LMP1*, *EBNA2*, and *EBNA3*. LMP1 and EBNA2 exert potent anti-apoptotic effects [35,36], while EBNA3 cooperates with EBNA2 to promote cell proliferation [37]. In contrast, EBV-positive gastric epithelial cells display type I latency and do not express LMP1, EBNA2, or EBNA3 [2]. In addition, dasatinib has been reported to increase expression of the chemokine receptor CXCR4 in LCLs [19]. However, RNA-seq analysis of dasatinib-treated AGS and AGS-EBV cells revealed no appreciable induction of CXCR4 expression in either cell type, with transcript levels remaining at background levels (TPM ≤ 0.05) under all conditions. These observations are consistent with the possibility that differences in host and viral gene expression profiles between LCLs and epithelial cells contribute to the epithelial cell–selective antitumor effects of dasatinib.

We previously reported that both vorinostat and dasatinib reduce BART miRNA promoter activity [5]. Vorinostat simultaneously suppresses BART miRNA expression and induces EBV lytic reactivation, consistent with reports showing that EBV strains lacking BART miRNAs exhibit increased BZLF1 expression and readily enter the lytic cycle [38]. In contrast, dasatinib treatment was associated with reduced BART miRNA expression (Figure 4A,B) together with reduced *BZLF1* promoter activity and lytic gene expression under the conditions tested (Figure 2A,B). Importantly, the present study does not establish a direct causal relationship between BART miRNA suppression and apoptosis induction; instead, these molecular events were observed in parallel following SFK inhibition under the experimental conditions examined.

Although dasatinib exerted a potent antitumor effect in EBV-positive epithelial cells, it did not show comparable activity in B lymphocytes. One possible explanation is that, in B lymphocytes, BZLF1 expression is initiated through SFK–ERK signaling downstream of the B-cell receptor [39,40], whereas EBV-positive epithelial cells lack B-cell receptor signaling. Another possibility is that EBV-positive epithelial cells express higher levels of BART miRNAs than B cells [4], such that suppression of BART miRNA expression by dasatinib may contribute to inhibition rather than induction of the lytic cycle. At present, the relative contribution of these potential mechanisms cannot be distinguished. Thus, dasatinib exhibits a molecular profile characterized by concurrent suppression of BART miRNA promoter activity and *BZLF1* promoter activity in EBV-positive epithelial cells under the experimental conditions examined.

Dasatinib reduced phosphorylation of SFKs and downstream ERK signaling, consistent with its established activity as a multi-target tyrosine kinase inhibitor. EBV-positive cells exhibited higher basal SFK activation than EBV-negative counterparts, suggesting that EBV infection creates a signaling environment that enhances sensitivity to SFK inhibition. Although LMP2A can activate Src signaling in B lymphocytes, our findings using LMP2A-deficient EBV indicate that dasatinib-induced apoptosis in gastric epithelial cells cannot be fully explained by inhibition of LMP2A-associated signaling alone. These observations suggest that EBV-associated epithelial signaling networks beyond LMP2A contribute to SFK activation and drug sensitivity.

A key observation of this study is that dasatinib suppresses BART miRNA expression in EBV-positive epithelial cells. BART miRNAs are known to promote cell survival by repressing pro-apoptotic host genes and maintaining latency-associated transcriptional programs. We observed that dasatinib reduced BART transcript levels while increasing expression of several apoptosis-related target genes, including *CASZ1a*, *OCT1*, *ARID2*, *TP53INP1*, and *DAB2*. These coordinated changes are consistent with a model in which suppression of viral miRNA expression relieves repression of host pro-apoptotic pathways.

Importantly, dasatinib suppressed *BZLF1* promoter activity and lytic gene expression while still inducing apoptosis, indicating that its pro-apoptotic effects occur independently of lytic reactivation. This feature distinguishes dasatinib from lytic induction strategies and suggests a mechanism of tumor cell elimination that does not promote viral replication.

The transcriptional intermediates linking SFK inhibition to suppression of the BART promoter remain to be defined. SFK signaling regulates multiple transcriptional pathways, including STAT3, AP-1, MYC, and NF-κB, which are implicated in EBV latency maintenance and oncogenic transcriptional programs. Attenuation of these signaling networks may contribute to reduced BART promoter activity; however, direct promoter occupancy and chromatin regulatory mechanisms require further investigation. Importantly, the present conclusions are supported by consistent findings across multiple EBV-positive epithelial models and independent mechanistic readouts.

In summary, we demonstrate that dasatinib preferentially induces apoptosis in EBV-positive gastric epithelial cells and is associated with coordinated suppression of SFK signaling and EBV-encoded BART microRNA expression. Because SFK activation has also been reported in Burkitt’s lymphoma, which exhibits type I EBV latency similar to EBVaGC [41], these findings raise the possibility that SFK-directed strategies may warrant evaluation in additional EBV-associated malignancies. Together, our findings reveal a previously underappreciated link between host tyrosine kinase signaling and viral microRNA regulation in EBV-infected epithelial cells and suggest that host kinase signaling may represent a context-dependent therapeutic vulnerability in EBV-associated epithelial malignancies.

## 5. Limitations

Several limitations of this study should be acknowledged. First, although dasatinib treatment was associated with coordinated suppression of SFK signaling, reduction in BART miRNA expression, and induction of apoptosis in EBV-positive gastric epithelial models, the present work does not establish the necessity or sufficiency of individual pathways. Genetic or rescue approaches will be required to define the precise causal relationships among SFK inhibition, viral microRNA regulation, and apoptotic induction.

Second, the mechanistic experiments were performed primarily in EBV-infected gastric epithelial cell models. Although we confirmed key observations in naturally EBV-positive gastric carcinoma cells, further validation across additional patient-derived EBV-positive models will be important to define the generalizability of these findings.

Third, the transcription factors and chromatin regulatory mechanisms linking SFK signaling to BART promoter activity remain to be elucidated. Future studies employing chromatin immunoprecipitation and promoter occupancy analyses will be necessary to define the transcriptional intermediates involved.

Fourth, although dasatinib induced apoptosis in EBV-positive cells irrespective of *TP53* mutation status, this study did not comprehensively address potential resistance mechanisms that may arise during long-term treatment. Elucidation of such mechanisms will be essential for future clinical applications.

Finally, while our results support a mechanism by which dasatinib selectively promotes apoptosis in EBV-positive epithelial cells, in vivo therapeutic efficacy and potential resistance mechanisms require further investigation before clinical translation can be considered.

## 6. Conclusions

This study provides evidence that dasatinib treatment is associated with preferential apoptosis in EBV-positive gastric epithelial cell models together with coordinated modulation of SFK signaling and BART miRNA expression. Unlike previously reported strategies that rely on induction of the EBV lytic cycle, dasatinib treatment was not accompanied by detectable productive lytic replication under the experimental conditions tested and was associated with apoptosis irrespective of *TP53* mutation status. Mechanistically, dasatinib treatment was associated with reduced BART miRNA levels, increased expression of several reported pro-apoptotic target genes, and decreased expression of anti-apoptotic factors such as BCL-XL and MCL1. These molecular changes occurred in parallel with apoptosis induction; however, further mechanistic studies will be required to determine their hierarchical relationships.

Collectively, these findings suggest that EBV BART miRNAs may represent a potential therapeutic vulnerability in EBV-associated epithelial malignancies and support further investigation of SFK-targeted approaches that modulate viral miRNA transcription without promoting lytic replication. The present work provides preclinical evidence supporting a functional link between host kinase signaling and EBV microRNA regulation, warranting further mechanistic and translational studies in EBV-associated cancers, including EBV-positive gastric carcinoma.

## Figures and Tables

**Table 1:** Regulatory effect of Dasatinib.

ID	Consistency Score	Node Total	Regulator Total	Regulators	Target Total	Target Molecules in Dataset	Disease & Function Total	Diseases & Functions	Known Regulator-Disease/Function Relationship
**1**	58.89	52	14	ATF4, CTNNB1, EDN1, ERBB2, ERK, F2, FOXO3, HGF, IFNG, Insulin, NFkB (complex), NRG1, RRAS2, Vegf	27	ARHGEF2, ASNS, ATF5, CCR3, CD274, DDIT3, EGFR, EGR1, EMP1, ERRFI1, ESM1, ETS1, FOSB, FOSL1, GLI1, GZMB, HBEGF, IER3, IL23A, KRT7, MAFF, NFE2L3, PPP1R15A, RELB, S100B, TAGLN, TRIB3	11	**Apoptosis of embryonic cell lines**, Cell cycle progression, Cell death of kidney cell lines, Cell movement of breast cancer cell lines, Cell proliferation of carcinoma cell lines, Cell proliferation of ovarian cancer cell lines, Expansion of lymphocytes, Invasion of cells, Invasive tumor, Production of reactive oxygen species, Transcription of RNA	69% (106/154)
**2**	52.746	69	18	ARNT2, CCND1, CTNNB1, ERK, F2, IL1A, IL5, KLF6, MYD88, NPM1, RAF1, RRAS2, SIM1, SMAD3, SMARCA4, TCF7L2, TICAM1, VEGFA	33	ARHGEF2, ASNS, CCR3, CD274, CREB5, DDIT3, ECM2, EGFR, EGR1, EMP1, ERRFI1, ESM1, ETS1, FOSB, FOSL1, GLI1, GZMB, HBB, HBEGF, IER3, IL23A, KRT5, KRT7, LGR6, MAFF, MAP1B, MYADM, PAEP, RELB, S100A3, S100B, TAGLN, UPP1	18	Activation of DNA endogenous promoter, Angiogenesis, Benign lesion, Cell cycle progression, Cell proliferation of carcinoma cell lines, Cell proliferation of ovarian cancer cell lines, Cell viability of lung cancer cell lines, Chemotherapy resistance of tumor cell lines, Expansion of lymphocytes, Growth of tumor, Invasion of cells, Invasive tumor, Migration of tumor cell lines, Perinatal death, Proliferation of lung cancer cell lines, Recruitment of leukocytes, Sensitivity of tumor cell lines, Transcription of RNA	47% (152/324)
**3**	51.008	63	19	ATF4, CTNNB1, EDN1, ERK, F2, HGF, IFNG, IL15, IL2, INS, Insulin, NFkB(complex), NUPR1, PDGF BB, RRAS2, SSTR2, TGFB1, Vegf, VEGFA	31	ARHGEF2, ASNS, CD274, CREB5, DDIT3, EGFR, EGR1, EMP1, ERRFI1, ESM1, ETS1, FOSB, FOSL1, GLI1, GZMB, HBEGF, IER3, IL23A, KRT13, KRT5, KRT7, LGR6, MAFF, MAP1B, NFE2L3, PPP1R15A, RELB, S100B, SIPA1L2, TAGLN, TRIB3	13	**Apoptosis of embryonic cell lines**, Benign lesion, Cell cycle progression, Cell death of kidney cell lines, Cell movement of breast cancer cell lines, Cell proliferation of ovarian cancer cell lines, Cell viability of lung cancer cell lines, Expansion of lymphocytes, Invasion of cells, Invasion of tumor, Invasive tumor, Migration of tumor cell lines, Perinatal death	50% (123/247)
**4**	49.391	58	16	ATF4, CG, CTNNB1, EDN1, ERBB2, ERK, F2, HGF, IFNG, IGF1, INS, Insulin, NFkB (complex), RB1, RRAS2, Vegf	31	ASNS, ATF5, CD274, CREB5, DDIT3, EGFR, EGR1, EMP1, ERRFI1, ESM1, ETS1, FOSB, FOSL1, GLI1, GZMB, HBEGF, IER3, IL23A, KRT13, KRT5, KRT7, LGR6, MAFF, NFE2L3, PHLDB2, PPP1R15A, RELB, S100B, TAGLN, TRIB3, UPP1	11	Benign lesion, Cell cycle progression, Cell death of embryonic cell lines, Cell death of kidney cell lines, Cell movement of breast cancer cell lines, Cell proliferation of carcinoma cell lines, Cell proliferation of ovarian cancer cell lines, Chemotherapy resistance of tumor cell lines, Expansion of lymphocytes, Invasion of cells, Invasive tumor	57% (101/176)
**5**	40.035	47	12	ARNT2, CTNNB1, ERK, F2, MYD88, PDGF BB, RRAS2, SIM1, SSTR2, TCF7L2, TICAM1, VEGFA	23	CD274, CREB5, DDIT3, EGFR, EGR1, ESM1, ETS1, FOSB, FOSL1, GLI1, GZMB, HBEGF, IER3, IL23A, KRT5, KRT7, LGR6, MAFF, PPP1R15A, RELB, S100B, TAGLN, TRIB3	12	Activation of DNA endogenous promoter, Angiogenesis, Benign lesion, Cell cycle progression, Cell movement of tumor cell lines, Cell proliferation of carcinoma cell lines, Cell proliferation of ovarian cancer cell lines, Invasion of cells, Invasive tumor, Migration of cells, Perinatal death, Transcription of RNA	52% (75/144)
**6**	33.586	61	17	ATF4, CTNNB1, EDN1, ERBB2, ERK, F2, HGF, IFNG, Insulin, NFkB (complex), NRG1, NUPR1, PDGF BB, RRAS2, TGFB1, Vegf, VEGFA	31	ARHGEF2, ASNS, ATF5, CCR3, CD274, CREB5, DDIT3, EGFR, EGR1, EMP1, ERRFI1, ESM1, ETS1, FOSB, FOSL1, GLI1, GZMB, HBEGF, IER3, IL23A, KRT5, KRT7, MAFF, MAP1B, NFE2L3, PPP1R15A, RELB, S100B, SIPA1L2, TAGLN, TRIB3	13	**Apoptosis of embryonic cell lines**, Cell cycle progression, Cell death of kidney cell lines, Cell movement of breast cancer cell lines, Cell proliferation of ovarian cancer cell lines, Cell viability of lung cancer cell lines, Expansion of lymphocytes, Invasion of cells, Invasive tumor, Migration of tumor cell lines, Perinatal death, Production of reactive oxygen species, Transcription of RNA	60% (133/221)
**7**	33.2	47	15	ATF4, CG, EDN1, EIF2AK3, ERBB2, ERK, ERK1/2, FOXO3, HGF, IFNG, IGF1, INS, Insulin, NFkB (complex), Vegf	25	ASNS, CD274, DDIT3, EGFR, EGR1, EMP1, ERRFI1, ESM1, ETS1, FOSB, FOSL1, GZMB, HBEGF, IER3, IL23A, KRT13, KRT7, MAFF, NFE2L3, PHLDB2, PPP1R15A, RELB, S100B, TAGLN, TRIB3	7	Benign lesion, Cell death of embryonic cell lines, Cell death of kidney cell lines, Cell movement of breast cancer cell lines, Expansion of lymphocytes, Invasion of cells, Invasive tumor	58% (61/105)
**8**	31.4	42	10	CTNNB1, EDN1, ERK, NPM1, NUPR1, PDGF BB, SMAD3, SSTR2, TGFB1, VEGFA	25	ARHGEF2, ASNS, CCR3, CD274, CREB5, DDIT3, EGFR, EGR1, ERRFI1, ETS1, FOSB, FOSL1, GLI1, GZMB, HBEGF, IER3, IL23A, KRT5, KRT7, LGR6, MAP1B, MYADM, PPP1R15A, RELB, TRIB3	7	Benign lesion, Cell cycle progression, Cell movement of tumor cell lines, Cell proliferation of ovarian cancer cell lines, Cell viability of lung cancer cell lines, Perinatal death, Recruitment of leukocytes	49% (34/70)
**9**	19.969	28	11	ATF4, CG, EDN1, EIF2AK3, ERBB2, ERK1/2, FOXO3, HGF, INS, Insulin, PDGF BB	13	DDIT3, EGFR, EGR1, EMP1, ERRFI1, ETS1, FOSB, FOSL1, IER3, KRT7, PPP1R15A, RELB, TRIB3	4	Benign lesion, Cell death of embryonic cell lines, Cell death of kidney cell lines, Cell movement of breast cancer cell lines	45% (20/44)
**10**	18.441	28	6	ATF4, CTNNB1, FOXO3, IL4, KLF6, RRAS2	14	ASNS, CD274, DDIT3, EGFR, EGR1, FOSL1, GLI1, HBEGF, IER3, KRT7, LGR6, PPP1R15A, RELB, TRIB3	8	**Apoptosis of embryonic cell lines**, Cell cycle progression, Cell death of kidney cell lines, Cell proliferation of ovarian cancer cell lines, Cell viability of lung cancer cell lines, Invasion of cells, Neoplasia of tumor cell lines, Proliferation of lung cancer cell lines	44% (21/48)
**11**	15.119	17	8	ATF4, CG, EIF2AK3, ERBB2, FOXO3, Insulin, P38 MAPK, TNF	7	DDIT3, EGFR, EGR1, EMP1, IER3, PPP1R15A, TRIB3	2	**Apoptosis of embryonic cell lines**, Cell death of kidney cell lines	69% (11/16)
**12**	7.757	11	4	IL1B, NFkB (complex), RB1, TNF	6	CD274, EGFR, EGR1, ETS1, IL23A, RELB	1	Expansion of blood cells	50% (2/4)
**13**	−6.5	6	1	UCP1	4	ARHGEF2, ASNS, ATF5, TRIB3	1	Cell proliferation of carcinoma cell lines	0% (0/1)
**14**	−6.5	6	1	UCP1	4	ARHGEF2, ATF5, DDIT3, TRIB3	1	Transcription of RNA	100% (1/1)
**15**	−7.506	5	1	HMG20A	3	HBEGF, TAGLN, TRIB3	1	Invasion of tumor cell lines	100% (1/1)
**16**	−7.506	5	1	Pka	3	DDIT3, EGR1, IL23A	1	Activation of DNA endogenous promoter	0% (0/1)
**17**	−8.083	5	1	TWNK	3	ASNS, ATF5, TRIB3	1	Cell proliferation of carcinoma cell lines	0% (0/1)

## Data Availability

The original contributions presented in this study are included in the article/Supplementary Materials. Further inquiries can be directed to the corresponding authors.

## Supplementary Materials

The following supporting information can be downloaded at: https://www.mdpi.com/article/10.3390/cancers18071082/s1, Figure S1: Dasatinib preferentially reduces viability in *TP53*-mutant EBV-positive epithelial cells; Figure S2: SFK inhibition preferentially reduces viability in EBV-positive epithelial cells; Figure S3: Dasatinib is associated with reduced lytic gene expression and viral DNA levels in *TP53*-mutant EBV-positive cells; Figure S4: Ponatinib reduces BART promotor activity and transcript levels; Figure S5: Dasatinib treatment is associated with increased expression of reported BART miRNA target genes.

## Abbreviations

The following abbreviations are used in this manuscript:

ARID2	AT-rich interaction domain 2
*BALF5*	*Bam*H I A leftward fragment 5
BART	*Bam*H I A rightward transcript
BCL-XL	B-cell lymphoma-extra-large
BCR-ABL	breakpoint cluster region-Abelson murine leukemia
*BLLF1*	*Bam*H I L leftward fragment 1
*BRLF1*	*Bam*H I R leftward fragment 1
*BZLF1*	*Bam*H I Z leftward fragment 1
CASZ1a	castor zinc finger 1a
cDNA	complementary DNA
CSK	C-terminal Src Kinase
DAB2	disabled homolog 2
DMSO	dimethyl sulfoxide
EBER	EBV encoded small RNA
EBNA1	EBV-associated nuclear antigen1
EBV	Epstein–Barr virus
EBVaGC	EBV-associated gastric carcinoma
eGFP	enhanced green fluorescent protein
EphA2	ephrin type-A receptor 2
ERK1/2	extracellular signal-regulated kinases 1/2
FBS	fetal bovine serum
GAPDH	glyceraldehyde 3-phosphate dehydrogenase
IC_50_	half-maximal inhibitory concentrations
KO	deficient
LCL	lymphoblastoid cell line
LMP2A	latent membrane protein 2A
MCL1	induced myeloid leukemia cell differentiation ptotein-1
OCT1	octamer-binding transcription factor 1
PD-L1	programmed cell death1-ligand 1
qPCR	quantitative polymerase chain reaction
RNA-seq	RNA Sequencing
RT-qPCR	reverse transcription–qPCR
SCID	Severe combined immunodeficiency
SFK	Src family kinases
TP53INP1	tumor protein p53-inducible nuclear protein 1
