# Supplementary material for: SFK Inhibition Suppresses EBV-Encoded BART miRNAs and Induces Apoptosis in EBV-Positive Gastric Epithelial Cells
Source: Cancers (Basel). 2026 Mar 26;18(7):1082. doi: 10.3390/cancers18071082 (PMC13072225; doi:10.3390/cancers18071082)
Supplement: Supplementary file 1 [file cancers-18-01082-s001.zip › Suppl. Fig rerevision.pptx]

## Slide 1
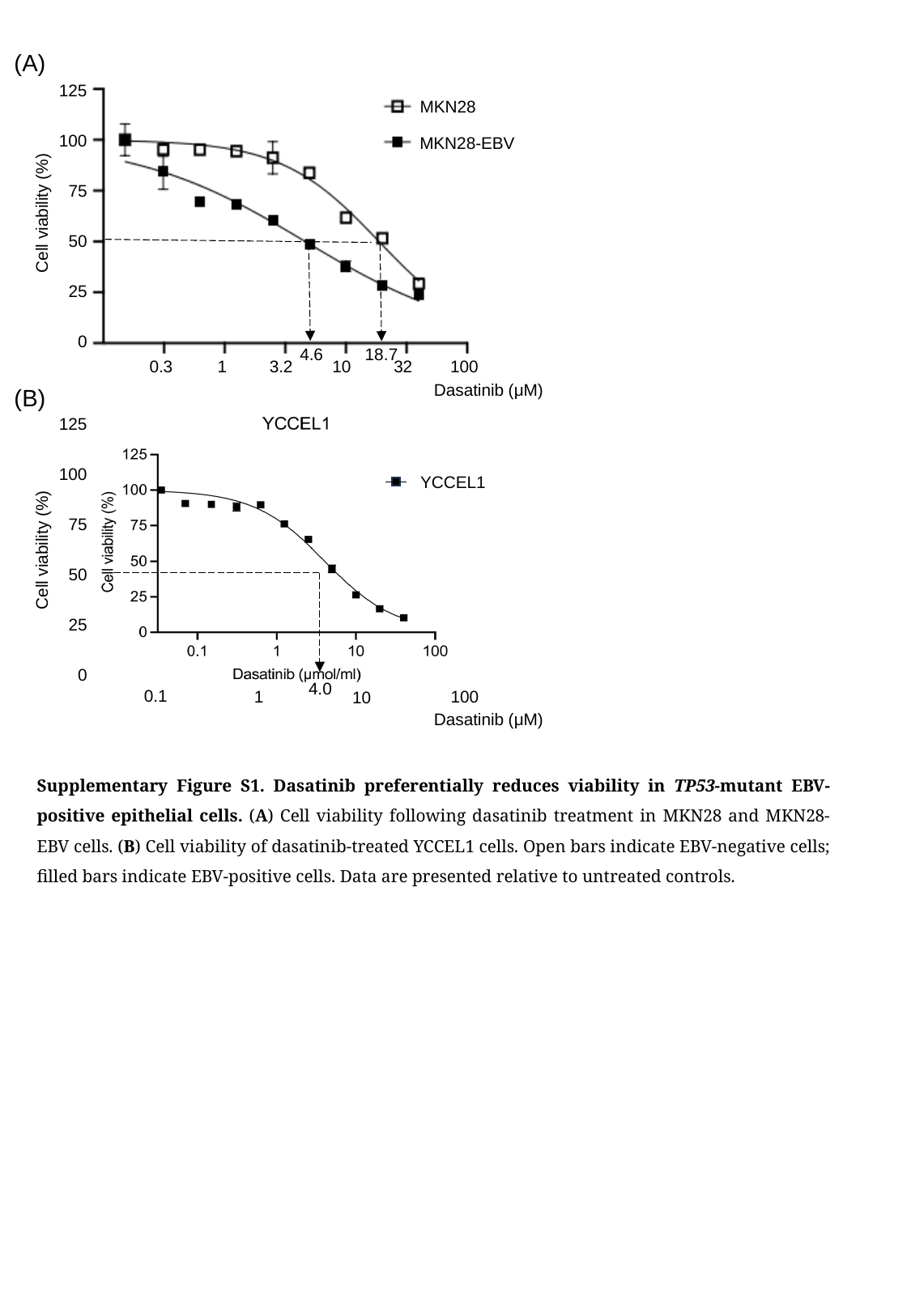

(A)
125
MKN28
100
MKN28-EBV
75
 Cell viability (%)
50
25
0
4.6 18.7
0.3
1
3.2
10
32
100
Dasatinib (μM)
(B)
125
100
YCCEL1
75
 Cell viability (%)
50
25
0
4.0
0.1
1
100
10
Dasatinib (μM)
Supplementary Figure S1. Dasatinib preferentially reduces viability in TP53-mutant EBV-positive epithelial cells. (A) Cell viability following dasatinib treatment in MKN28 and MKN28-EBV cells. (B) Cell viability of dasatinib-treated YCCEL1 cells. Open bars indicate EBV-negative cells; filled bars indicate EBV-positive cells. Data are presented relative to untreated controls.

## Slide 2
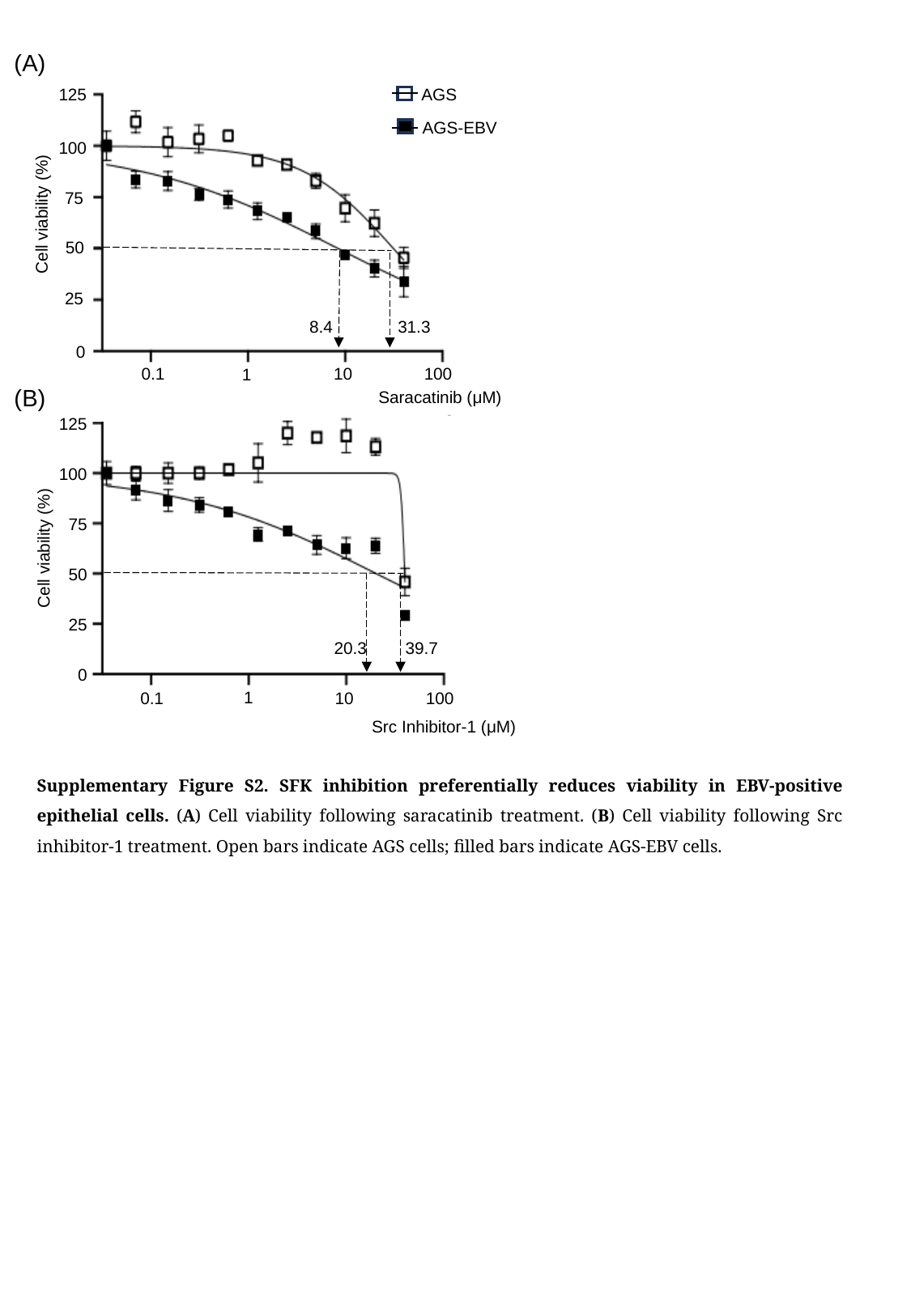

(A)
125
AGS
AGS-EBV
100
75
Cell viability (%)
50
25
8.4
31.3
0
0.1
10
100
1
(B)
Saracatinib (μM)
125
100
75
 Cell viability (%)
50
25
20.3
39.7
0
1
0.1
100
10
Src Inhibitor-1 (μM)
Supplementary Figure S2. SFK inhibition preferentially reduces viability in EBV-positive epithelial cells. (A) Cell viability following saracatinib treatment. (B) Cell viability following Src inhibitor-1 treatment. Open bars indicate AGS cells; filled bars indicate AGS-EBV cells.

## Slide 3
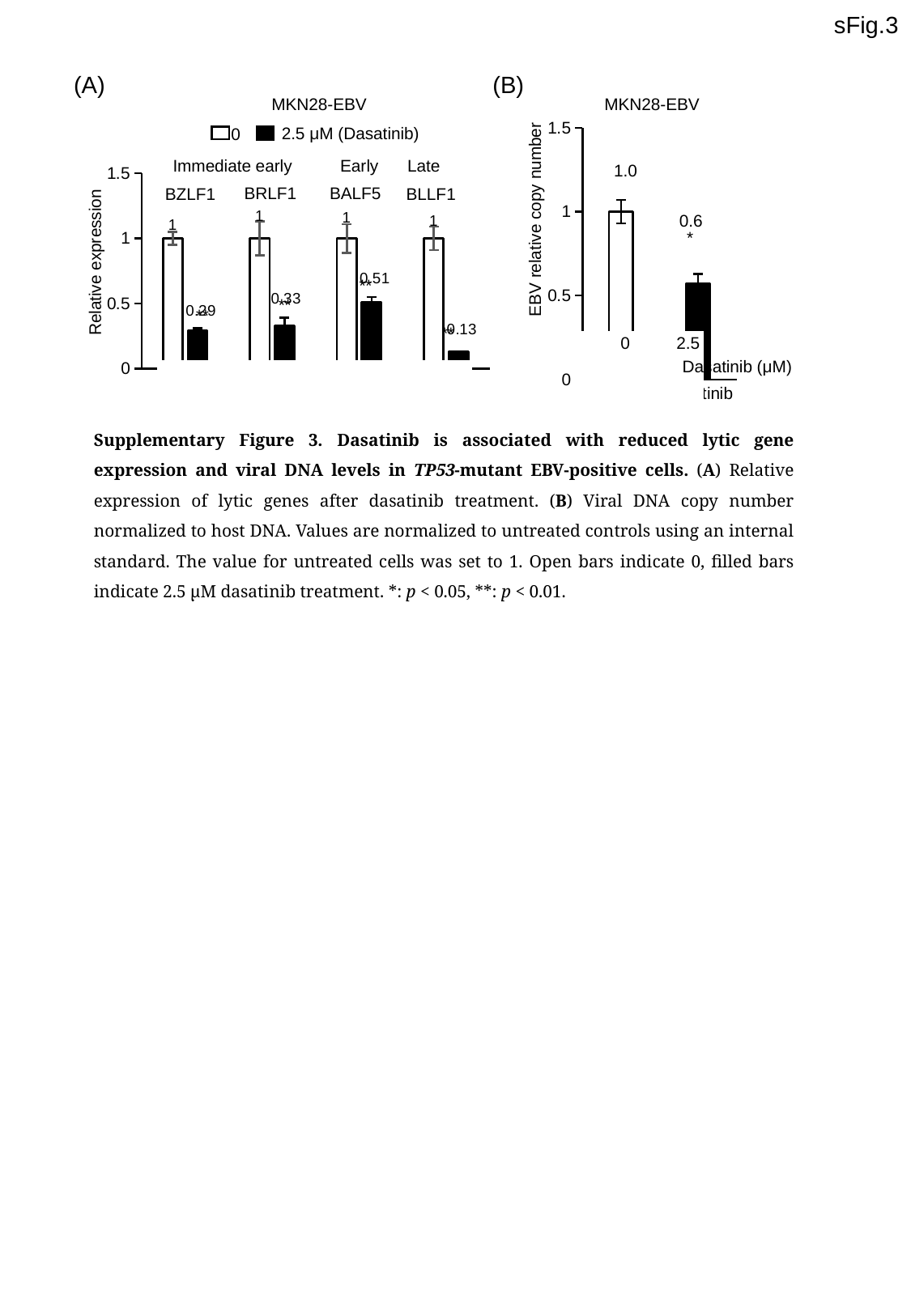

sFig.3
(A)
(B)
MKN28-EBV
MKN28-EBV
### Chart
| Category | |
|---|---|
| Control | 1.0 |
| Dasatinib | 0.5705049610859226 |2.5 μM (Dasatinib)
0
Late
Immediate early
Early
1.0
### Chart
| Category | | |
|---|---|---|BALF5
BRLF1
BZLF1
BLLF1
EBV relative copy number
0.6
*
Relative expression
**
**
**
**
 0 2.5
Dasatinib (μM)
Supplementary Figure 3. Dasatinib is associated with reduced lytic gene expression and viral DNA levels in TP53-mutant EBV-positive cells. (A) Relative expression of lytic genes after dasatinib treatment. (B) Viral DNA copy number normalized to host DNA. Values ​​are normalized to untreated controls using an internal standard. The value for untreated cells was set to 1. Open bars indicate 0, filled bars indicate 2.5 μM dasatinib treatment. *: p < 0.05, **: p < 0.01.

## Slide 4
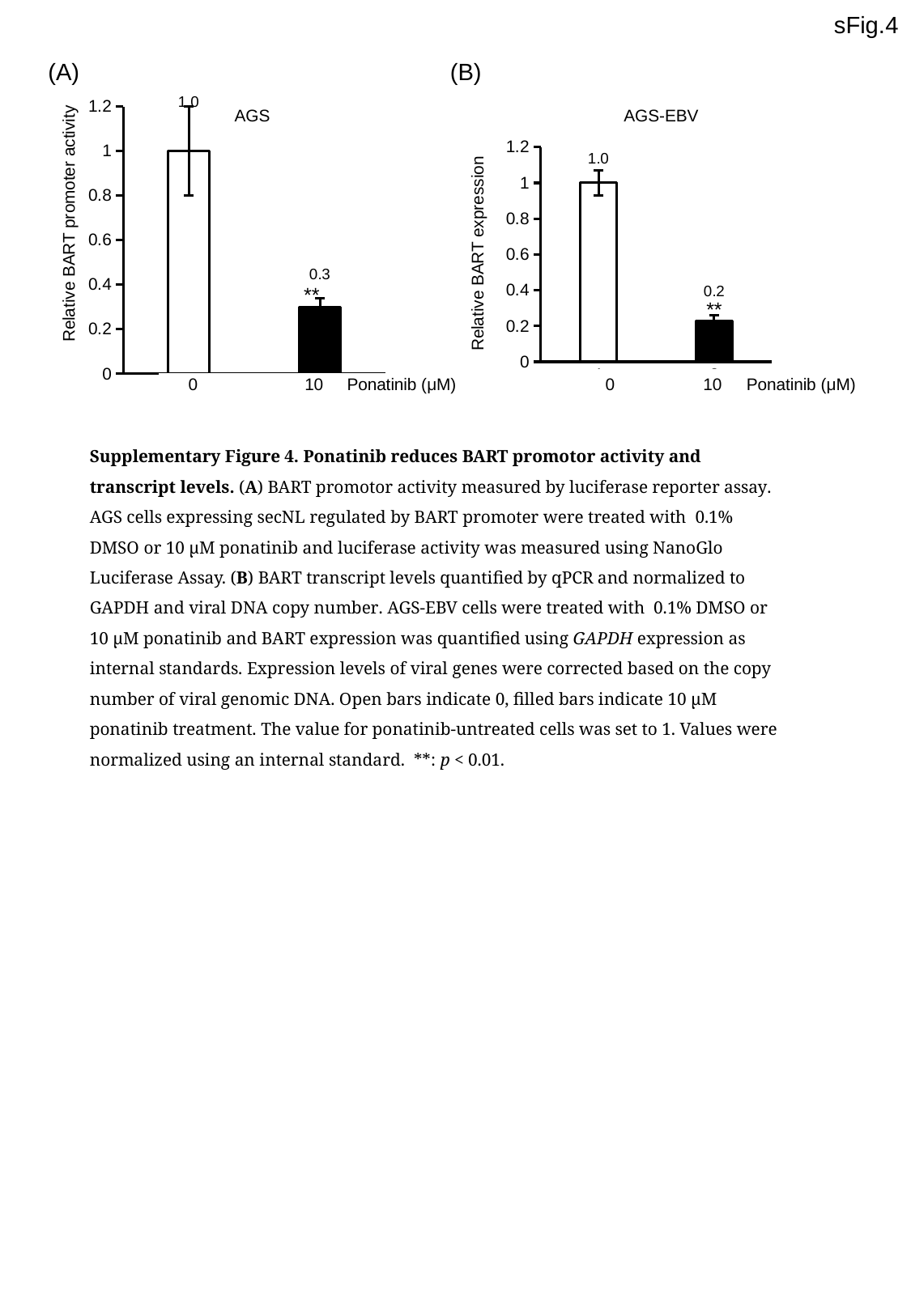

sFig.4
(A)
(B)
### Chart
| Category | |
|---|---|AGS
AGS-EBV
### Chart
| Category | |
|---|---|
Relative BART promoter activity
Relative BART expression
**
**
0 10
Ponatinib (μM)
0 10
Ponatinib (μM)
Supplementary Figure 4. Ponatinib reduces BART promotor activity and transcript levels. (A) BART promotor activity measured by luciferase reporter assay. AGS cells expressing secNL regulated by BART promoter were treated with 0.1% DMSO or 10 μM ponatinib and luciferase activity was measured using NanoGlo Luciferase Assay. (B) BART transcript levels quantified by qPCR and normalized to GAPDH and viral DNA copy number. AGS-EBV cells were treated with 0.1% DMSO or 10 μM ponatinib and BART expression was quantified using GAPDH expression as internal standards. Expression levels of viral genes were corrected based on the copy number of viral genomic DNA. Open bars indicate 0, filled bars indicate 10 μM ponatinib treatment. The value for ponatinib-untreated cells was set to 1. Values ​​were normalized using an internal standard. **: p < 0.01.

## Slide 5
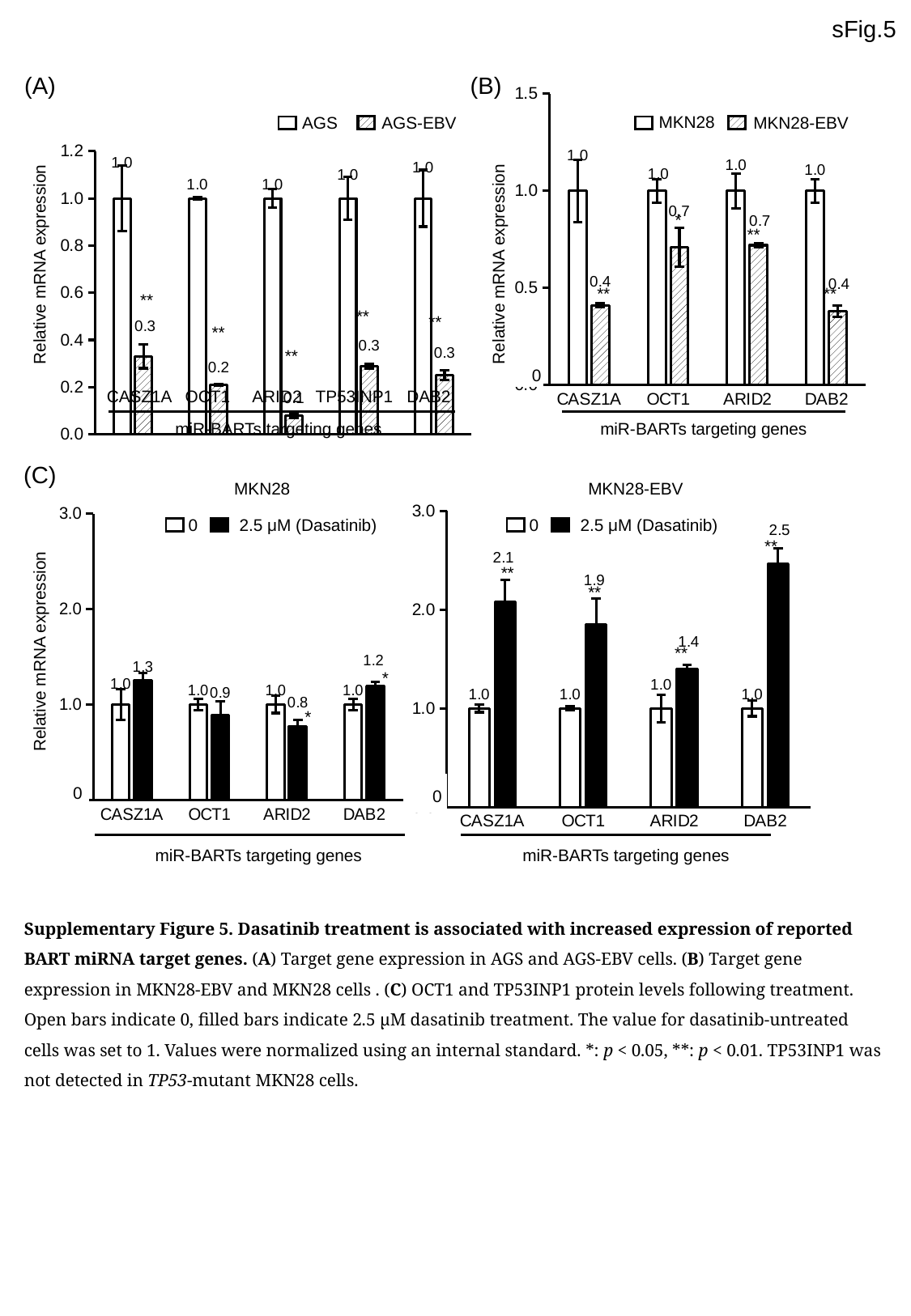

sFig.5
(A)
(B)
### Chart
| Category | | |
|---|---|---|
| CASZ1A | 1.0 | 0.41 |
| OCT1 | 1.0 | 0.71 |
| ARID2 | 1.0 | 0.72 |
| DAB2 | 1.0 | 0.38 |MKN28
MKN28-EBV
AGS
AGS-EBV
### Chart
| Category | | |
|---|---|---|
| CASZ1a | 1.0 | 0.33 |
| OCT1 | 1.0 | 0.21 |
| ARID2 | 1.0 | 0.08 |
| TP53INP1 | 1.0 | 0.29 |
| DAB2 | 1.0 | 0.25 |*
**
Relative mRNA expression
Relative mRNA expression
**
**
**
**
**
**
**
0
CASZ1A OCT1 ARID2 TP53INP1 DAB2
miR-BARTs targeting genes
miR-BARTs targeting genes
(C)
MKN28
MKN28-EBV
### Chart
| Category | | |
|---|---|---|
| CASZ1A | 1.0 | 1.25 |
| OCT1 | 1.0 | 0.89 |
| ARID2 | 1.0 | 0.77 |
| DAB2 | 1.0 | 1.19 |
### Chart
| Category | | |
|---|---|---|
| CASZ1A | 1.0 | 2.08 |
| OCT1 | 1.0 | 1.85 |
| ARID2 | 1.0 | 1.4 |
| DAB2 | 1.0 | 2.46 |0
2.5 μM (Dasatinib)
0
2.5 μM (Dasatinib)
**
**
**
Relative mRNA expression
**
*
*
0
0
miR-BARTs targeting genes
miR-BARTs targeting genes
Supplementary Figure 5. Dasatinib treatment is associated with increased expression of reported BART miRNA target genes. (A) Target gene expression in AGS and AGS-EBV cells. (B) Target gene expression in MKN28-EBV and MKN28 cells . (C) OCT1 and TP53INP1 protein levels following treatment. Open bars indicate 0, filled bars indicate 2.5 μM dasatinib treatment. The value for dasatinib-untreated cells was set to 1. Values ​​were normalized using an internal standard. *: p < 0.05, **: p < 0.01. TP53INP1 was not detected in TP53-mutant MKN28 cells.
