# Supplementary material for: SFK Inhibition Suppresses EBV-Encoded BART miRNAs and Induces Apoptosis in EBV-Positive Gastric Epithelial Cells
Source: Cancers (Basel). 2026 Mar 26;18(7):1082. doi: 10.3390/cancers18071082 (PMC13072225; doi:10.3390/cancers18071082)
Supplement: Supplementary file 1 [file cancers-18-01082-s001.zip › Western original data.pptx]

## Slide 1
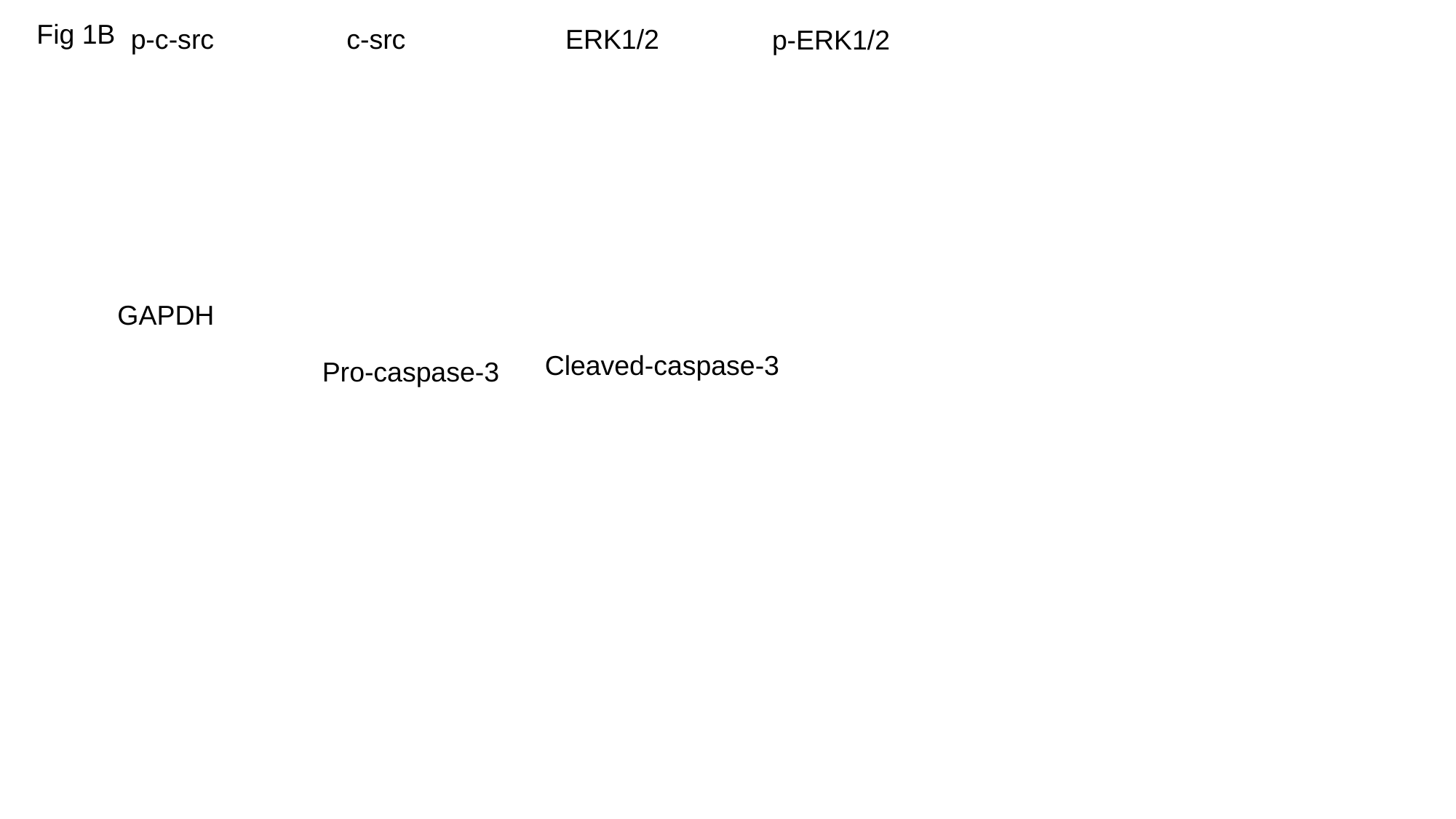

Fig 1B
p-c-src
c-src
ERK1/2
p-ERK1/2
GAPDH
Cleaved-caspase-3
Pro-caspase-3

## Slide 2
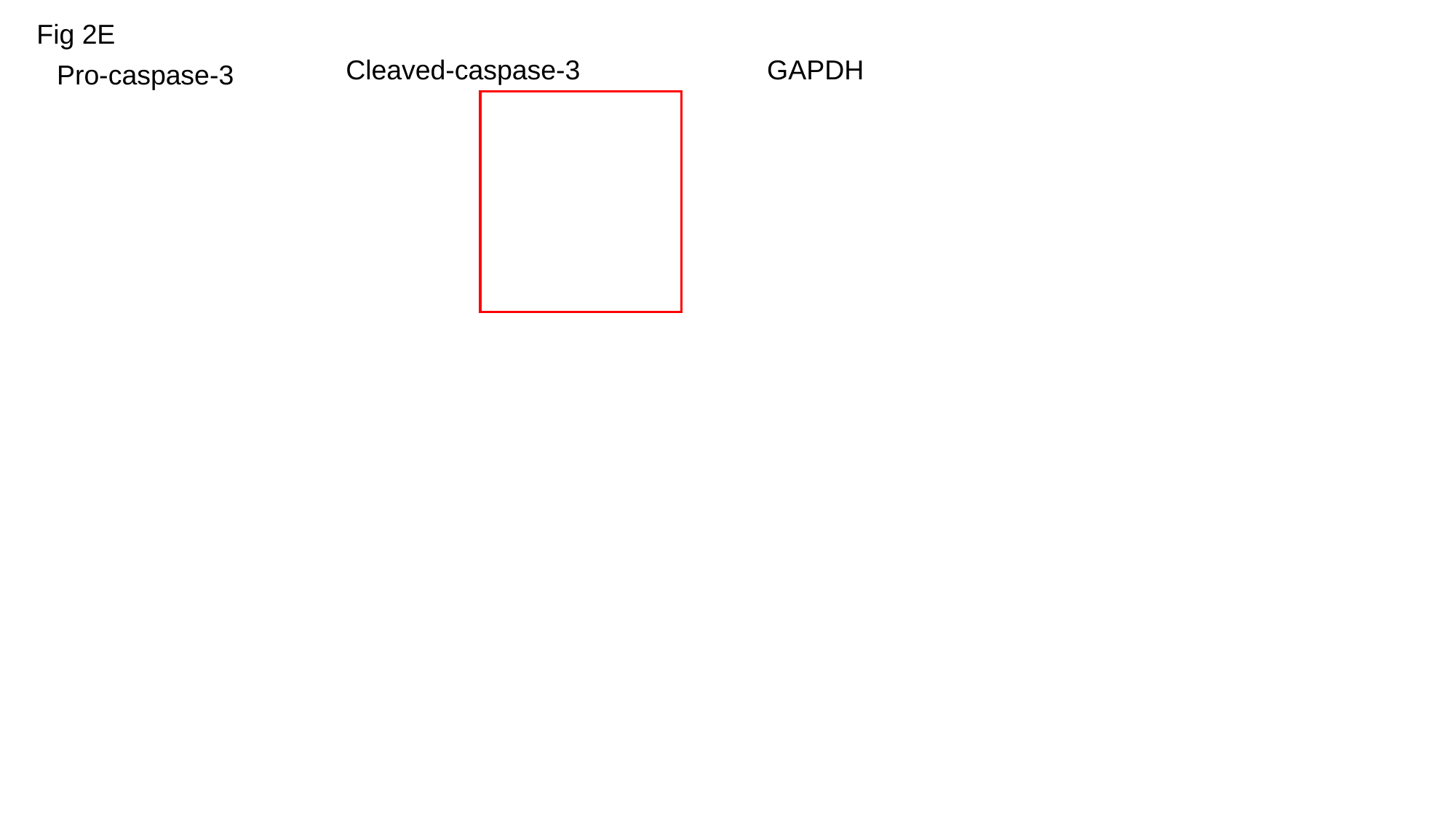

Fig 2E
Cleaved-caspase-3
GAPDH
Pro-caspase-3

## Slide 3
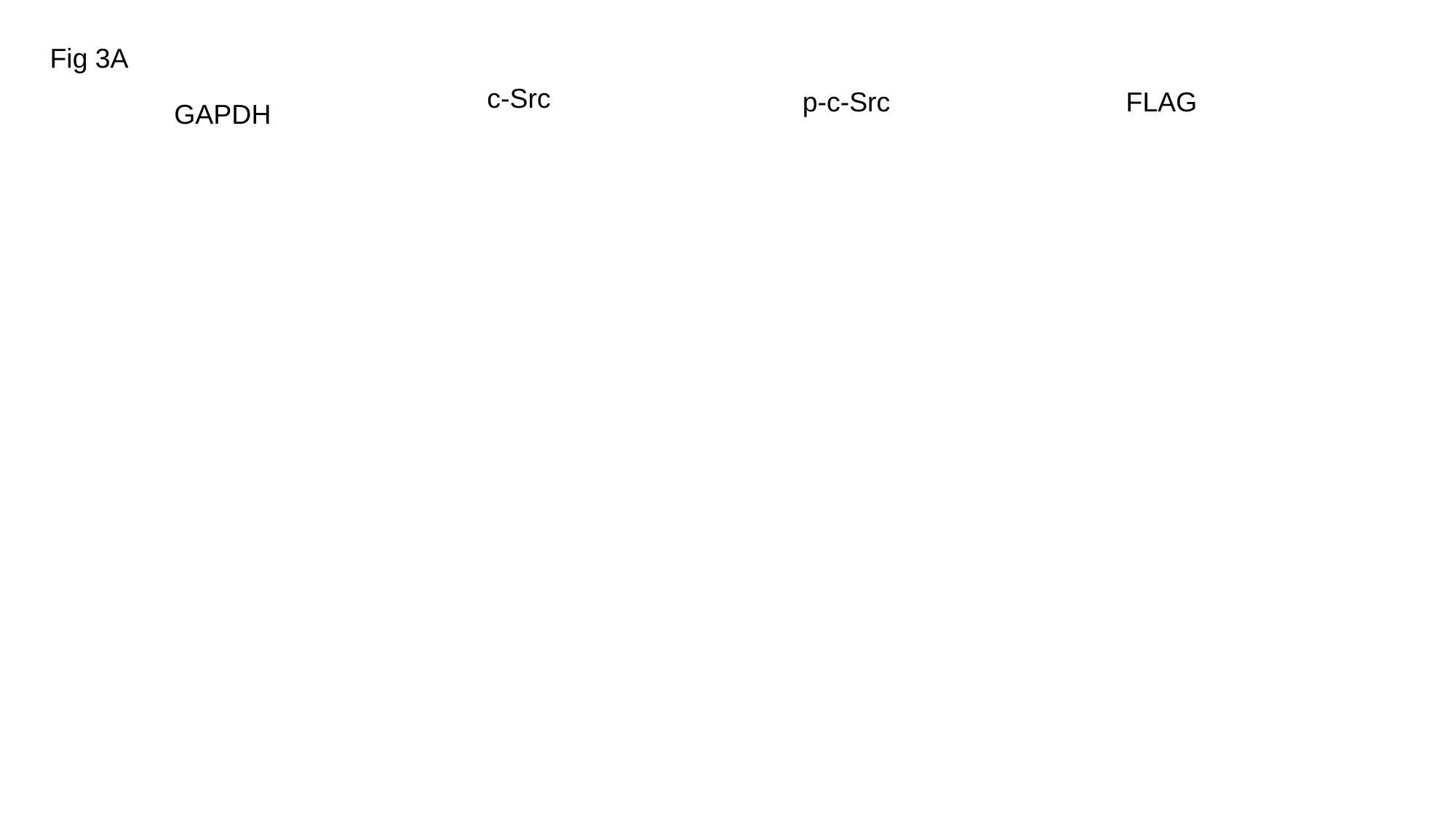

Fig 3A
c-Src
p-c-Src
FLAG
GAPDH

## Slide 4
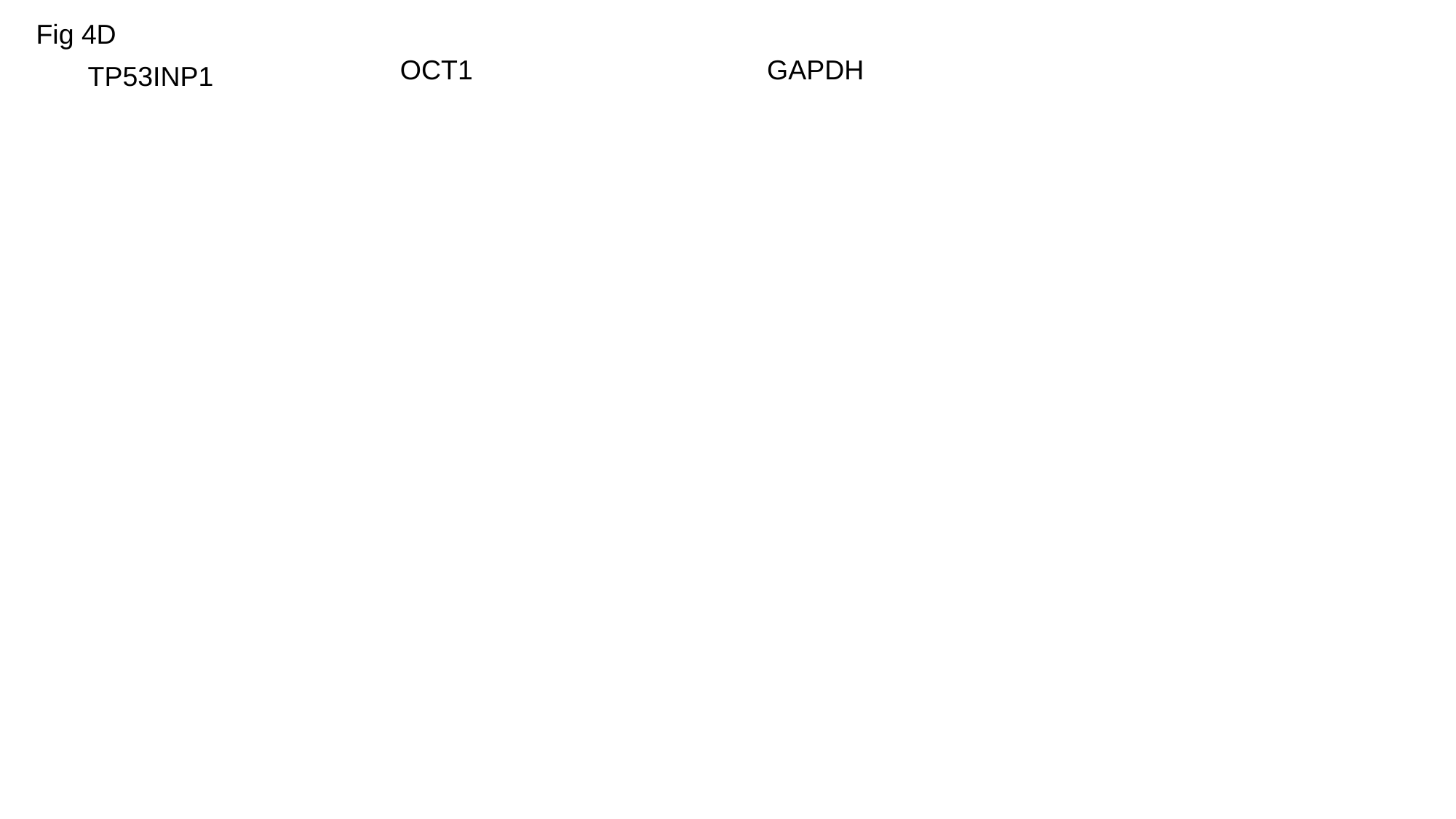

Fig 4D
OCT1
GAPDH
TP53INP1
